# *In Silico*-Based High-Throughput Screen for Discovery of Novel Combinations for Tuberculosis Treatment

**DOI:** 10.1128/AAC.05148-14

**Published:** 2015-08-14

**Authors:** Ragini Singh, Vasanthi Ramachandran, Radha Shandil, Sreevalli Sharma, Swati Khandelwal, Malancha Karmarkar, Naveen Kumar, Suresh Solapure, Ramanatha Saralaya, Robert Nanduri, Vijender Panduga, Jitendar Reddy, K. R. Prabhakar, Swaminathan Rajagopalan, Narasimha Rao, Shridhar Narayanan, Anand Anandkumar, V. Balasubramanian, Santanu Datta

**Affiliations:** a Cellworks Research India Pvt. Ltd., Bengaluru, India; b AstraZeneca India Pvt. Ltd., Bengaluru, India

## Abstract

There are currently 18 drug classes for the treatment of tuberculosis, including those in the development pipeline. An *in silico* simulation enabled combing the innumerably large search space to derive multidrug combinations. Through the use of ordinary differential equations (ODE), we constructed an *in silico* kinetic platform in which the major metabolic pathways in Mycobacterium tuberculosis and the mechanisms of the antituberculosis drugs were integrated into a virtual proteome. The optimized model was used to evaluate 816 triplets from the set of 18 drugs. The experimentally derived cumulative fractional inhibitory concentration (∑FIC) value was within twofold of the model prediction. Bacterial enumeration revealed that a significant number of combinations that were synergistic for growth inhibition were also synergistic for bactericidal effect. The *in silico*-based screen provided new starting points for testing in a mouse model of tuberculosis, in which two novel triplets and five novel quartets were significantly superior to the reference drug triplet of isoniazid, rifampin, and ethambutol (HRE) or the quartet of HRE plus pyrazinamide (HREZ).

## INTRODUCTION

Tuberculosis (TB) caused by Mycobacterium tuberculosis continues to be a major global health problem, with an estimated 8.6 million new cases and 1.3 million deaths reported in 2012 (1). The African and Southeast Asian regions contributed about 57% of all new TB cases. Among all new cases, an estimated 450,000 people developed multidrug-resistant (MDR) TB, and an estimated 170,000 deaths from MDR-TB occurred. This problem is further accentuated by the high incidence of coinfection of TB patients with the human immunodeficiency virus (HIV). The current first-line treatment is failing, and drug resistance is emerging rapidly in all regions of the world. The need of the hour is to discover novel regimens that are synergistically effective and act within a shortened duration of therapy (2, 3). The current therapy for drug-sensitive tuberculosis recommended by WHO, termed DOTS (directly observed treatment short course), is a combination of four drugs, *viz*., rifampin (RIF), isoniazid (INH), pyrazinamide (PZA), and ethambutol (EMB) (4). Despite the WHO guidelines (5), treatment modes have been chaotic for individuals with MDR or extensively drug resistant (XDR) TB in countries like India (6). Physicians are often forced to choose among the available antitubercular agents depending on the patient's disease and financial status, the cost of drugs, and the tolerability profile (7, 8). Since most of these alternatives have poor tolerability and are moderately effective at best, the treatment outcomes are hardly encouraging.

The use of multidrug combinations is a given paradigm in the treatment of tuberculosis. While there are guidelines in terms of how future combination regimens should be derived (9), the drug discovery cascade and preclinical development center to a large extent on the progression of individual drugs through these processes. At a late stage in discovery, combinations are typically tried based on the convenience of sourcing and what is topical at the moment. Our approach of employing a computational model offers the ability for the drug developer to remain agnostic to the drugs to be included in a combination along with a new one that is being developed, and this can happen very early in the discovery cascade, thus opening up better opportunities to discover an optimal combination rather than settle for what appears feasible.

With the understanding of how the ebb and flow of metabolites is related to the growth and death of bacteria, we have built an *in silico* dynamic network of 15 interlinked pathways that were chosen based on their connection to the central carbon metabolism and their potential for possessing drug targets; for example, enoyl-acyl carrier protein (ACP) reductase for INH or DNA gyrase for fluoroquinolones. This platform elicits responses to perturbations that are similar to the way the bacteria respond in the real world. The details of such a model for Escherichia coli were published previously (10). In this study, we report the development of an *in silico* model for M. tuberculosis based on ordinary differential equations (ODE) to identify novel synergistic combinations. We mapped the inhibitory reactions of 18 TB drugs (see the supplemental material), including compounds in clinical development, based on published literature. They are INH (11–14), RIF (15), EMB (16–18), amikacin (AMK) (19), streptomycin (STR) (20), kanamycin (KAN) (21, 22), capreomycin (CAP) (23, 24), clarithromycin (CLR) (25), moxifloxacin (MXF) (26, 27), meropenem (MEM) (28, 29), d-cycloserine (DCS) (30, 31), clofazimine (CFZ) (32), thiacetazone (THI) (33), bedaquiline (BDQ; previously TMC207) (34, 35), linezolid (LZD) (36), PA824 (pretomanid) (37), SQ109 (38), and BTZ043 (39). Hence, to derive a 4-drug combination, one would employ the formula 18C4 (combinations without repetitions), which is 3,060 combinations that describe the quartet space for 18 drugs. Assuming each drug/compound is tested across 6 different concentrations (or dosages) in order to derive the optimum synergistic or even additive dose, the total number of test conditions would be 3,060 × 6 × 6 × 6 × 6 = 3,965,760. This large search space is obviously not testable in an experimental format. However, with the aid of the *in silico* platform, we have studied the combinatorial search space, following which a prioritized list of combinations were studied *in vitro* for superior bactericidal effect. Subsequently, a further shortlist of bactericidal combinations were tested *in vivo* in a chronic model of tuberculosis in mice. This triage resulted in the identification of several combinations that were superior to the standard first-line regimen in the mouse model.

## MATERIALS AND METHODS

### Bacterial strains, growth conditions, and chemicals.

M. tuberculosis H37Rv ATCC 27294, a strain susceptible to all standard anti-TB drugs, was used for all of the studies in this report. The inoculum used for all experiments was derived from a seed lot maintained at −70°C that was prepared after a single round of broth amplification of bacilli isolated from infected mouse lungs.

The antituberculosis drugs used in this study were procured from commercial sources or synthesized to order. INH, RIF, PZA, streptomycin sulfate, ethambutol dihydrochloride, kanamycin B sulfate, amikacin hydrate, CFZ, CLR, CAP, THI, and DCS were procured from Sigma Chemical Co., USA. MEM was provided by AstraZeneca Pharmaceuticals, United Kingdom. MXF, PA824, BDQ, SQ109, BTZ043, and LZD were purchased from Wuxi Apptec, China.

M. tuberculosis H37Rv was grown in 250-ml roller bottles (Corning) as smooth cultures to mid-log phase (optical density at 600 nm [OD_600_] of 0.5) and stored frozen as 0.5-ml aliquots in screw-cap cryovials (Corning) at −70°C. Representative vials from the frozen lot were thawed and plated for viable counts after 10 days and were found to contain ∼10^8^ CFU/ml. For subsequent experiments, seed lot vials were thawed, and the cells were diluted to get 3 × 10^5^ to 5 × 10^5^ CFU/ml. The media used for growth of M. tuberculosis were Middlebrook 7H9 broth and 7H10 agar (Difco Laboratories) supplemented with 0.2% glycerol, 0.05% Tween 80, and 10% albumin-dextrose-catalase (ADC).

### Animals.

All experimental protocols involving animals and the use of animals were approved by the Institutional Animal Ethics Committee, registered with the Government of India (registration no. CPCSEA 1999/5). The BALB/c mice used for these studies were 6 to 8 weeks old with an average body weight of 20 to 25 g (RCC, Hyderabad). Mice were randomly assigned to cages and allowed to acclimatize for 2 weeks prior to experiments. Feed and water were given ad libitum.

### *In silico* platform.

In order to understand how perturbations are generated and transmitted within the bacterial cell due to inhibition of specific enzymes that are housed in interconnected cellular pathways, we built an *in silico* platform through the use of major M. tuberculosis pathways. Our *in silico* M. tuberculosis system is a dynamic network of 15 interlinked pathways, including glycolysis, the pentose phosphate pathway, the tricarboxylic acid (TCA) cycle along with the glyoxylate shunt, fatty acid metabolism (FAS I and FAS II system), biosynthesis of branched-chain amino acids, pantothenic acid, and coenzyme A (CoA), cell wall pathways (mycolic acid, arabinogalactan, and peptidoglycan synthesis), the nicotinamide biosynthesis pathway, and replication, transcription, and translation machinery. These pathways were chosen based on their connection to central carbon metabolism and their potential for possessing drug targets that are distributed across various interconnecting pathways. Modeling of the replication machinery ensured dynamic changes to DNA, which is a major component of the biomass. With a view to having comprehensive coverage of physiological factors that influence gene expression, we included transcription factors, activators, and inhibitors, as well as protein formation by translational regulators. Thus, the roles of transcription and translation machinery in enzyme synthesis were also modeled. Expanding the transcription and translation machinery gave the leverage to capture the effects of various perturbations that may possibly feed in at the gene expression and protein levels. This also helped to expand the drug coverage to targets like RNA polymerase and gyrases. Published data on enzyme kinetics (*K_m_*, *V*_max_, and *K_i_*), pathway flux distribution, operon structure, and gene regulation were used to build the *in silico* platform. The kinetics of enzymatic and pathway functioning was simulated by interconnecting ODE describing the kinetic behavior of each enzyme in the pathway. A kinetic model was thus constructed within the computational and mathematical framework by using intracellular enzyme concentrations and other kinetic parameters, such as *V*_max_ and *K_m_*. In cases where the kinetic data were not available, the data were reengineered by using a proprietary algorithm that aligned predicted data sets to published data under various growth and inhibition conditions.

### *In silico* monitoring of growth arrest.

Each drug exerts its inhibitory effect on growth via a certain mechanism of action (MOA). Thus, a drug's action in the *in silico* platform is simulated by affecting the target genes/pathways that are implicated in its MOA. The effects of various combinations are then monitored with respect to synergy, indifference, or antagonism. Since the marker “biomass arrest” is common to both kinds of antibiotics (bactericidal and bacteriostatic) and is also the one that is routinely evaluated in the laboratory as defined by the MIC, we used this as the marker of growth arrest in our synergy simulation.

After integrating the various pathways as described above, the final product from each pathway was assimilated into a biomass reaction, which included macromolecules like protein, DNA, lipids, etc., with the known stoichiometry obtained from the literature (40, 41). The biomass equation is a representation of M. tuberculosis cell growth. A typical simulation at *t* = 0 s begins with zero biomass, then a significant lag in the curve is observed where the cell is synthesizing the precursor molecules, followed by the rapid increase during log phase, and finally, stationary phase. The mycobacterial cell growth is simulated for 172,800 s (48 h). Upon the introduction of an antibiotic during the simulation, e.g., MXF, which inhibits *gyrA*, the growth curve is terminated, resulting in a plateau of the biomass curve, simulating the arrest of the growth of the organism.

The biomass generated in the *in silico* platform is given by the following equation: 0.214 × MTB_protein + 0.036 × MTB_RNA + 0.022 × MTB_DNA + 0.01 × MTB_PE + 0.05 × MTB_Sm_Mol + 0.02 × MTB_sugar + 0.01 × MTB_DMPP + 0.17 × MTB_Poly_l_Glu + MTB_Myc_AG_PG + 0.0080 × MTB_LAM + 0.09 × MTB_AC1_PIM4 + 0.02 × MTB_TAG = MTB_BM, where MTB_protein is the total protein content of the cell, MTB_RNA is the total RNA content of the cell, MTB_DNA is the total DNA content of the cell, MTB_PE is the phosphatidylethanolamine, MTB_Sm_Mol is the summation of all small molecules produced within the cell, such as NAD, etc., MTB_sugar is the sugars, MTB_DMPP is dimethylallyl pyrophosphate, MTB_Poly_l_Glu is poly-l-glutamine, MTB_Myc_AG_PG is mycolic acid arabinogalactan peptidoglycan complex (cell wall), MTB_LAM is lipoarabinomannan, MTB_AC1_PIM4 is phosphatidyl inositol mannosides, MTB_TAG is tri-acyl-glycerol, and MTB_BM is the total biomass.

### Analysis of synergy and antagonism.

When two drugs with independent MOAs are administered, their pathways interact, resulting in indifference, antagonism, or synergism. *In vitro* combinations are usually assessed on the basis of the fractional inhibitory concentration (FIC) index, which represents the sum of the FICs of each drug tested, where the FIC is determined for each drug by dividing the MIC of each drug when used in combination by the MIC of each drug when used alone (42). The equation above is true in the case of *n* ≥ 2 drugs acting in combination, which is expressed as follows: ∑FIC = FIC_*A*_ + FIC_*B*_ + FIC_*C*_ + ….FIC_*N*_, where FIC_*A*_ is the FIC of drug A, etc.

### Reverse engineering of *K_i_* in the *in silico* model.

The MICs are defined as the lowest concentration of an antimicrobial agent that will inhibit the visible growth of a microorganism after overnight incubation (43). The binding kinetics of a drug to its target and the effect on the function of the target define how the drug molecule interferes with the bacterial physiology to produce a therapeutic response. This is referred to as the mechanism of action (MOA) of the drug. MOAs influence clinical efficacy, safety, and duration of action and differentiate medicines. With the experimentally derived MIC for a given drug, the *K_i_* in the platform at its site of action is modulated until arrest of biomass is observed in the platform. This modulation is done with the help of reverse-engineering techniques, where different values of *K_i_* are tested in the *in silico* model. Then, an optimal *K_i_* value is chosen such that a higher value would show survival of the mycobacterium and a lower value would show growth arrest or inhibition.

### Computational synergy module.

With the reverse-engineered *K_i_* for the drugs, combinations of two drugs were simulated in order to propose the kinds of interaction that they share. In order to present the interactive relationship, isobolograms were used. The MIC of each drug in the combination was considered 1 unit. In our *in silico* simulation, the drug is added when bacterial growth reaches steady state. This is *t* = 0 s in our simulation. The total run time for a wild-type uninhibited cell is 172,800 s (48 h). In the drug simulation runs, the effect on biomass is seen as a plateauing of the biomass curve. To begin with, the first combination was checked, where 0.5× MIC of drug A [*f*(*x*) of A, where *x* = 0.5] and 0.5× MIC of drug B [*f*(*y*) of B, where *y* = 0.5] were considered. The combination was simulated using a simulator developed in-house, and the results were analyzed as follows:

### (i) Survival of the organism.

This fact states that the combination of drug concentrations was not adequate to arrest biomass generation in M. tuberculosis.

### (ii) Inhibition of the organism.

This fact states that the combination of drug concentrations was adequate to arrest biomass generation in M. tuberculosis.

If the cell survives, then it is concluded that the additive concentration is not enough and the dose needs to be increased to arrest the growth of M. tuberculosis. In such a scenario, *I* = *f*(*x*) + *f*(*y*) > 2, which corresponds to an antagonistic combination.

If the growth is arrested at the concentration being considered, the dose is lowered and the organism checked for survival. Either simultaneous reduction of the fractional concentrations of both drugs or reduction of the concentration of each drug one by one is done, followed by analysis of the results. If the organism survives the previous data point, the value is retained for plotting the isobole; otherwise, the dose is lowered further. These data points mimic *I* = *f*(*x*) + *f*(*y*) < 0.5, referring to a synergistic combination. Data points are also found to lie in the zone of additivity, i.e., *f*(x) + *f*(*y*) is >0.5 and <2; such a situation is referred to as an additive combination. Taking into account the twofold (one well) experimental variability, additivity is depicted by the zone between 0.5 and 2.0 instead of the line where *f*(*x*) + *f*(*y*) = 1.0.

The *in silico* model was validated using pairwise combinations. Among the entire list of 153 combinations that are possible (18C2), 65 combinations (∼40%) were tested *in vitro*, which is an adequate representation of the entire set.

### *In vitro* MIC determination.

The MICs of antibiotics against M. tuberculosis H37Rv were determined using the resazurin-based microplate assay (44).

### *In vitro* two-dimensional (2-D) checkerboard analysis of antimicrobial inhibition.

Serial double dilutions of drug A and drug B were prepared in rows (B to G) and columns (2 to 11), respectively, in a 96-well microplate. M. tuberculosis H37Rv culture (200 μl at 10^5^ cells/ml) was dispensed into all wells except those in column 1, which was used as a no-growth control. Column 12 did not have antibiotics and was used as a growth control. The assay plates were incubated at 37°C for 6 days, and growth was monitored using resazurin dye, as mentioned above. The fractional inhibitory concentration (FIC) was calculated as the ratio of the MIC in combination with the MIC of a single agent. The cumulative fractional inhibitory index (∑FIC) was calculated as the sum of the FICs of drug A and drug B to evaluate interaction profiles. A ∑FIC of <0.5 was interpreted as synergism, a ∑FIC of >2 as antagonism, and values in between as additive.

### *In vitro* three-dimensional (3-D) checkerboard analysis of antimicrobial inhibition.

For studying triplet combinations, the protocol described above was followed to initially set up the checkerboard assay with doublets in multiple plates. The third drug was introduced at different concentrations but at one concentration per plate. The remaining steps were followed as described above for the 2-D checkerboard analysis.

### *In vitro* bactericidal-effect analysis.

For determining the extent of the bactericidal effect, sample wells which remained blue in the resazurin microtiter assay (REMA) and whose ΣFICs were within the synergistic-to-additive range (<0.7 for doublets and <1.5 for triplets) were plated on Middlebrook 7H10 agar medium and incubated at 37°C for 21 to 28 days, following which bacterial colonies were enumerated to obtain the net reduction in CFU in comparison to the counts for the untreated controls at the start of drug testing, as well as to that achieved when M. tuberculosis was exposed to each drug alone.

### *In vivo* efficacy study.

BALB/c mice were infected with approximately 100 bacilli per mouse by using the Madison aerosol chamber. Infected mice were housed in individually ventilated cages (Allentown Technologies) in a biosafety level 3 (BSL3) facility. A total of 20 treatment groups were included in the study, excluding the pretreatment and posttreatment control groups which received the vehicle alone. Treatment was initiated 4 weeks after the onset of infection and was administered orally once a day, 6 days a week, for 4 weeks. Forty-eight hours after the completion of treatment, mice were euthanized with CO_2_ and their lungs harvested and homogenized in phosphate-buffered saline (PBS) containing 0.1% bovine gelatin and 0.1% Triton X-100 using tissue grinders (catalog no. W012576; Wheaton). Each suspension was serially diluted in 10-fold steps and plated on Middlebrook 7H11 agar supplemented with 10% albumin-dextrose-catalase. The plates were incubated at 37°C with 5% CO_2_ for 3 weeks, and CFUs enumerated. Dunnett's multiple-comparison test was used to assess differences in lung CFUs in treated versus untreated mice.

### *In vivo* pharmacokinetic studies.

The drugs were formulated in 0.5% hydroxypropyl methyl cellulose (HPMC)–0.1% Tween 80 suspensions as 3- or 4-drug combinations. Formulations were prepared by weighing and adding each compound to the bottle, according to the design. Formulation vehicle was added, and the suspension was stirred overnight. Only uniform suspensions were used, while those that precipitated were abandoned. Formulations were prepared every week, and compound stability was estimated at the end of the week. Pharmacokinetics (PK) analysis was performed on infected mice on the 20th day of dosing. Blood samples were collected from each mouse at 0.5, 2, 4, 6, and 24 h after compound administration. Blood samples of about 30 μl were collected from mice in all groups by serial sampling via the saphenous vein into Microvette CB300 tubes (Starstedt, Germany) coated with EDTA, and plasma (10 μl) was separated following centrifugation. Plasma samples were stored at –20°C until analysis using liquid chromatography-tandem mass spectrometry (LC-MS/MS).

### Bioanalysis of plasma samples for estimating drug concentration.

A 1-mg/ml stock solution of each compound was prepared in dimethyl sulfoxide (DMSO) for all of the analytes except PZA and LZD, for which the stock solution was 10 mg/ml in DMSO, and RIF, for which the stock solution was prepared in 50% acetonitrile in water. Three different sets of calibration standard (CS) combinations were prepared by 2-fold dilution of the stock solutions. CS set 1 contained analytes CLO, BDQ, PA824, PZA, and SQ109, CS set 2 contained analytes BTZ043, MXF, THI, EMB, and PZA, and CS set 3 contained LZD, INH, RIF, and PZA. A 16-point calibration curve was utilized for all analytes, and the standard curves ranged from 0.0005 to 20 μg/ml except for PZA and LZD, for which the standard curve range was 0.006 to 200 μg/ml. Plasma samples were precipitated by adding chilled acetonitrile (1:10, vol/vol) containing carbamazepine as an internal standard (10 ng/ml). Samples were vortexed and then centrifuged at 4,000 rpm for 30 min at 10°C. The resulting supernatant was mixed with the mobile phase (50% acetonitrile in water with 0.1% formic acid). Five microliters of the sample was injected on to a liquid chromatographic system (Acquity ultraperformance liquid chromatography [UPLC] system, Waters, Milford, MA) coupled to a triple-quadrupole mass spectrometer (Xevo TQ-S; Waters, Milford, MA). Samples were separated on an LC column (ACE 3 C_18_, 50 by 4.6 mm, 3 μm particle size; Advanced Chromatography Technologies, Aberdeen, Scotland) by isocratic elution with 40 parts of 20 mM ammonium formate containing 0.1% (vol/vol) formic acid and 60 parts of acetonitrile at a flow rate of 0.5 ml/min with a run time of 4 min. Samples were acquired in positive ion mode and detected by multiple reaction monitoring (MRM). The concentrations of the analyte were determined from a standard curve obtained by plotting known concentrations of the analyte against peak area ratios (analyte/internal standard peak response).

### PK data analysis.

PK analyses of the plasma concentration-time relationships were performed with Phoenix software (version 6.2; Pharsight, USA). A noncompartmental analysis program, model 200, was used to calculate PK parameters. The maximum concentration of drug in plasma (*C*_max_), time to *C*_max_ (*T*_max_), elimination half-life (*t*_1/2_), and area under the concentration-time curve (AUC) from time zero to infinity (AUC_0–∞_) were estimated. AUC was computed using the trapezoidal rule (linear up and log down), and the AUC_0–∞_ was considered only when the extrapolated AUC was not more than 20% of the original value. A minimum of three sample points in the terminal slope were used to calculate half-life.

## RESULTS

An *in silico* M. tuberculosis platform that is capable of simulating the effects of drugs singly or in combination was built by interconnecting ordinary differential equations that describe the kinetic behavior of enzymes in the various biochemical pathways. This is a dynamic network of 15 interlinked pathways that include central carbon metabolism, amino acid and cell wall biosynthesis, and core features like replication, transcription, and translation. These pathways were chosen based on their central roles in cellular growth and survival and their potential for possessing drug targets. We had earlier built a similar platform for E. coli. Since we had to overcome the paucity of kinetic data for M. tuberculosis enzymes, we reengineered the parameters by using a proprietary algorithm that aligned predicted data sets to published data under various growth conditions and a large set of MIC data for various antitubercular agents in stand-alone and doublet combination mode.

We monitored growth inhibition in the platform according to significant decreases in biomass levels. Each drug exerts its inhibitory effect via a certain mechanism of action (MOA). Thus, the action of a drug in the *in silico* platform is simulated by affecting the target genes/pathways that are implicated in its MOA. We mapped the inhibition by 18 drugs in the *in silico* platform (see “Drug mechanism incorporation in the *in silico* platform” in the supplemental material). The effects of various combinations were then monitored with respect to synergy, indifference, or antagonism. Since the biomass arrest marker is common to both kinds of antibiotics (bactericidal and bacteriostatic) and is also the one that is routinely evaluated in the laboratory as defined by MIC, we used this as the marker of growth arrest in our synergy simulation. The experimentally derived MIC and the reverse engineered *K_i_* as inferred from the *in silico* platform are shown in Table 1.

**TABLE 1:** MICs from the *in vitro* assay, CFU count at the MIC for each drug in the *in vitro* assay, and reverse-engineered *K_i_* values from the *in silico* platform

Drug	Code	MIC (μM)	*K_i_* (μM)	ΔLog_10_ CFU/ml at the MIC
Amikacin	AMK	0.43	18	−0.20
BTZ043	BTZ043	0.0009	7.75E−06	1.40
Capreomycin	CAP	5.91	132	−0.11
Clarithromycin	CLR	9.63	380	0.75
Clofazimine	CFZ	0.26	0.1	0.72
d-Cycloserine	DCS	30.59	0.01	0.87
Ethambutol	EMB	19.61	0.2	1.40
Isoniazid	INH	0.22	0.002	1.02
Kanamycin	KAN	1.61	70	0.44
Linezolid	LZD	2.31	101	0.25
Meropenem	MEM	8.15	0.001	0.57
Moxifloxacin	MXF	0.02	0.01	1.40
PA824	PA824	1.09	0.1	1.07
Rifampin	RIF	0.009	0.1	1.24
SQ109	SQ109	1.18	0.0002	1.40
Streptomycin	STR	0.68	29	0.36
Thiacetazone	THI	8.47	0.0002	0.76
Bedaquiline	BDQ	0.35	0.2	0.02
Pyrazinamide^*a*^	PZA	ND^*b*^	ND	ND

a PZA was included in the *in vivo* studies only.

b ND, the MIC and associated values were not determined in the present study.

**FIG 1:**
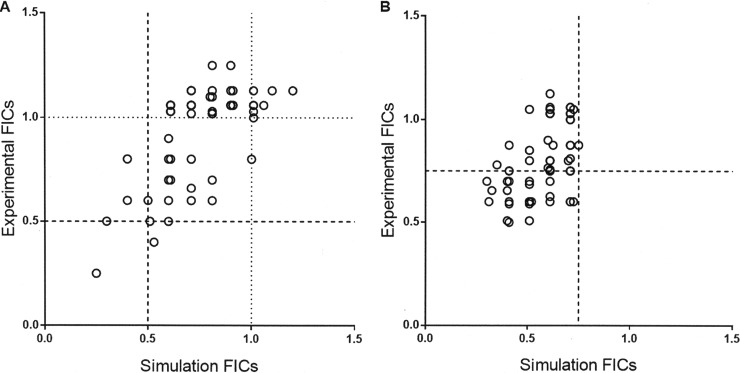
Correlation of FIC results from *in silico* versus *in vitro* studies. (A) 2-D combination studies; 65 doublets were analyzed. (B). 3-D combination studies; 51 triplets were analyzed. The experimental values were within twofold of the predicted value.

The individual drug actions of known antituberculosis agents were simulated, followed by testing the pairwise and triplet combinations. The platform was validated by testing pairwise combinations with a standard *in vitro* checkerboard method. A total of 153 pairwise combinations that are possible from 18 drugs (18C2) were studied in the *in silico* platform. Of these, 65 pairwise combinations were studied *in vitro* in order to validate the results from the *in silico* platform. The best ∑FICs across the 65 pairs were within twofold of each other when compared between the *in silico* and *in vitro* test systems (Fig. 1A; see also Fig. S1 in the supplemental material). The interday reproducibility across *in vitro* experiments was established, which enabled batch mode testing of combinations, since all combinations could not be tested in a single experiment (see Fig. S2).

Subsequently, 816 triplet combinations (18C3) were studied in the platform. A three-step process of filtration that removed the additive and indifferent combinations was done prior to selecting 55 triplet combinations that were then studied *in vitro* for ∑FIC, as well as bactericidal effect. This was necessitated by the fact that an exhaustive computational analysis over the 10-concentration range for each drug in a given triplet would have required 1,000 simulations per triplet and a total of 816,000 simulations. With the available in-house grid-computing infrastructure, this would have consumed 13 h per triplet, totaling 442 days for the 816 triplets. In the first step for all 816 triplets, a set of 13 equally fractionated concentrations, from FIC_*A*_ 0.1 + FIC_*B*_ 0.1 + FIC_*C*_ 0.1 to FIC_*A*_ 1 + FIC_*B*_ 1 + FIC_*C*_ 1, were tested in the model, thus yielding ∑FICs from 0.3 to 3. Of these, 360 triplets that resulted in the inhibition of growth at a ∑FIC of <1.2 were advanced for an exhaustive analysis of synergy. In the second step, among the 360 triplets, the exhaustive concentration range for any given triplet was decided by the outcome of the first step. In the case of 2 triplets where growth inhibition was seen at a ∑FIC of 0.3, a concentration range involving FICs of 0.025, 0.05, 0.75, and 0.1 was carried out, resulting in 4 × 4 × 4 = 64 simulations per triplet, totaling 128 simulations. In the case of 58 triplets where growth inhibition was seen at a ∑FIC of 0.6, the range was extended to include a FIC of 0.2 for each drug, resulting in 125 simulations per triplet × 58 triplets = 7,250 simulations. In the case of 130 triplets where growth inhibition was seen at a ∑FIC of 0.9, this was extended to include a FIC of 0.3 for each drug, resulting in 216 × 130 = 28,080 simulations. Finally, in the case of 170 triplets where growth inhibition was seen at a ∑FIC of 1.2, this was extended to include a FIC of 0.4 for each drug, resulting in 343 × 170 = 58,310 simulations. Thus, for 360 triplets, the total number of simulations was reduced to 93,768 simulations, resulting in a 90% decrease in overall time without any sacrifice in the accuracy of the data. The top 51 of these triplets were studied in 3-D checkerboard *in vitro* studies. As with the pairwise combinations, the predictive power of the simulation was highly significant, with only a 2.5-fold deviation between experimental FICs and simulation FICs (Fig. 1B; see also Fig. S3 and Table S1 in the supplemental material).

The synergy obtained with the 3-drug combinations was also tested for bactericidal effect by plating for CFU enumeration. Table 1 shows the extent of kill observed for each drug when tested at its MIC, i.e., a FIC of 1. One of the most significant findings in this report is that only some of the combinations that were synergistic for the growth inhibition based on FIC analysis were also synergistic for killing M. tuberculosis. From the 3-D checkerboard experiments, only those test wells which were at the interface of growth and no-growth were plated. Such an interface corresponds to the minimum concentration that resulted in the inhibition of growth as observed in the REMA. The data in Table 2 (see also Table S2 in the supplemental material) show the extent of bacterial kill obtained in the case of each triplet at its most potent synergistic ∑FIC index value, where the individual drug concentrations were at FICs of ≤0.5, i.e., half their MICs. Based on reductions of ≥2 Log_10_ CFU/ml, 31/51 triplets tested were synergistic for bactericidal effect. The best ∑FIC for growth inhibition did not always yield the most bactericidal combination. This was a key finding in this study, because there were synergistic combinations based on ∑FIC that were less bactericidal than the individual drugs. For example, BTZ043, MXF, and SQ109 are individually bactericidal, but when combined yield only stasis at a synergistic ∑FIC of 0.75. This indicates antagonism at the level of bactericidal effect, even though there is synergy for growth inhibition. Another example is the combination of CFZ, MXF, and THI.

**TABLE 2:** *In vitro* bactericidal analysis of 51 triplet drug combinations along with the reference regimen of INH, RIF, and EMB^*a*^

Drug:			FIC*_A_*	FIC*_B_*	FIC*_C_*	∑FIC index	ΔLog_10_ CFU/ml
A	B	C					
AMK	EMB	THI	0.5	0.5	0.125	1.125	−0.3
AMK	MEM	MXF	0.125	0.5	0.125	0.75	1.44
BTZ043	CAP	MEM	0.125	0.25	0.5	0.875	1.52
BTZ043	CFZ	LZD	0.125	0.5	0.125	0.75	1.87
BTZ043	EMB	SQ109	0.5	0.125	0.125	0.75	3.7
BTZ043	KAN	MEM	0.125	0.125	0.5	0.75	1.42
BTZ043	MEM	BDQ	0.125	0.5	0.125	0.75	1.38
BTZ043	MXF	SQ109	0.125	0.5	0.125	0.75	3.2
BTZ043	MXF	THI	0.125	0.5	0.25	0.875	0.7
BTZ043	PA824	BDQ	0.125	0.125	0.5	0.75	3.44
BTZ043	SQ109	THI	0.125	0.5	0.125	0.75	2.7
CLR	SQ109	THI	0.125	0.5	0.125	0.75	2.7
CFZ	EMB	PA824	0.125	0.5	0.25	0.875	0.3
CFZ	EMB	SQ109	0.5	0.125	0.125	0.75	1.63
CFZ	EMB	THI	0.5	0.125	0.5	1.125	0.25
CFZ	LIN	BDQ	0.25	0.125	0.5	0.875	0.27
CFZ	MXF	SQ109	0.5	0.25	0.5	1.25	0.7
CFZ	MXF	THI	0.125	0.125	0.5	0.75	0.65
CFZ	PA824	SQ109	0.125	0.25	0.5	0.875	3.7
CFZ	PA824	THI	0.5	0.125	0.125	0.75	3.4
CFZ	PA824	BDQ	0.125	0.5	0.5	1.125	2.06
CFZ	SQ109	THI	0.125	0.5	0.125	0.75	2.7
DCS	EMB	SQ109	0.125	0.5	0.5	1.125	3.7
DCS	EMB	THI	0.5	0.5	0.125	1.125	1.28
DCS	MXF	SQ109	0.5	0.5	0.5	1.5	0.3
DCS	MXF	THI	0.5	0.5	0.125	1.125	0.3
DCS	PA824	BDQ	0.125	0.5	0.25	0.875	2.92
DCS	SQ109	THI	0.125	0.5	0.5	1.125	2.7
EMB	LZD	SQ109	0.5	0.125	0.5	1.125	2.19
EMB	LZD	THI	0.5	0.5	0.25	1.25	0.27
EMB	SQ109	MXF	0.5	0.5	0.125	1.125	3.7
EMB	MXF	THI	0.5	0.5	0.125	1.125	0.3
EMB	PA824	SQ109	0.125	0.25	0.5	0.875	3.7
EMB	PA824	THI	0.125	0.25	0.25	0.625	3.7
EMB	PA824	BDQ	0.5	0.125	0.5	1.125	2.16
EMB	SQ109	THI	0.125	0.25	0.5	0.875	2.7
EMB	SQ109	BDQ	0.125	0.5	0.25	0.875	2.62
EMB	THI	BDQ	0.125	0.125	0.5	0.75	2.24
KAN	SQ109	THI	0.125	0.5	0.25	0.875	2.7
LZD	MXF	SQ109	0.5	0.5	0.125	1.125	0.25
LZD	MXF	THI	0.25	0.125	0.5	0.875	0.89
LZD	SQ109	THI	0.5	0.5	0.25	1.25	1.85
MEM	SQ109	THI	0.125	0.5	0.125	0.75	2.7
MXF	PA824	SQ109	0.125	0.125	0.5	0.75	3.4
MXF	PA824	THI	0.125	0.25	0.25	0.625	3.7
MXF	PA824	BDQ	0.25	0.25	0.5	1	3.1
MXF	SQ109	THI	0.5	0.5	0.125	1.125	3.4
PA824	SQ109	THI	0.125	0.5	0.125	0.75	3.7
PA824	SQ109	BDQ	0.5	0.125	0.25	0.875	2.7
STR	SQ109	THI	0.25	0.125	0.5	0.875	2.7
SQ109	THI	BDQ	0.5	0.25	0.125	0.875	2.8
INH	RIF	EMB	0.5	0.5	0.5	1.5	−0.3

a The maximum bacterial kill achieved for each combination with each drug concentration not greater than 0.5 FIC, i.e., 1/2 MIC, is shown.

The *in vivo* efficacy in the chronic model of tuberculosis in BALB/c mice was determined for nine test triplets and their associated quartets that included PZA. We included PZA in the *in vivo* studies because of its reported role in sterilization *in vivo*, even though we did not study the combinations that included PZA either in the *in silico* platform or in the *in vitro* studies. The *in vitro* combination studies with PZA were not carried out because it requires an acidic pH for testing *in vitro*, which renders the testing incompatible with other agents. Since *in vitro* validation was not possible, the *in silico* model with PZA was also not carried out.

In the *in vivo* study, the plasma exposures for the drugs in the present combination study were similar to their individual exposures, except for SQ109 and EMB (45–50). Exposure to SQ109 was higher in all the combinations than in previously reported data when tested individually. The observed AUC for SQ109 in combinations (2 to 8.9 μg · h/ml) was higher than the reported AUC (0.25 μg · h/ml) at a dose of 25 mg/kg of body weight. On the contrary, the AUC for EMB in combinations (4 to 9.9 μg · h/ml) was lower than its reported AUC (28 μg · h/ml) at a 100-mg/kg dose (see Table S3 in the supplemental material).

**FIG 2:**
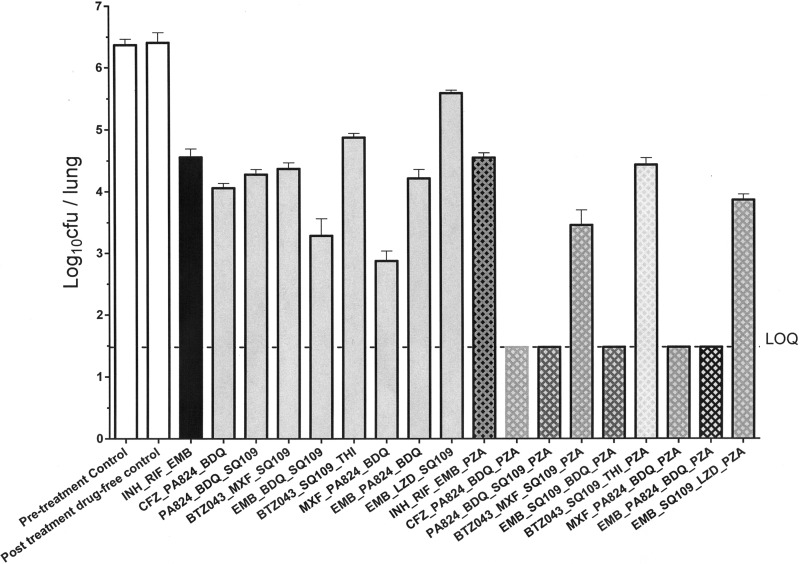
*In vivo* efficacy of triplets and quartets in a chronic model of tuberculosis in BALB/c mice following aerosol infection. LOQ, limit of quantitation (30 CFU/ml of lung homogenate).

**FIG 3:**
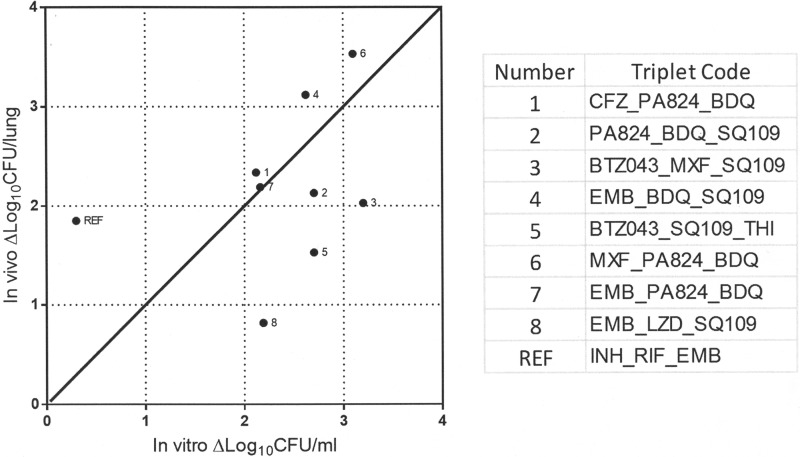
*In vitro-in vivo* correlation of bactericidal effect of combinations. The maximum ΔLog_10_ CFU/ml achieved with each drug concentration that was not greater than 0.5 FIC, i.e., 1/2 MIC, in the combination from the *in vitro* experiment (see Table S4 in the supplemental material) was plotted against the ΔLog_10_ CFU/lung from the *in vivo* experiment.

The triplets were compared to the INH-RIF-EMB combination, whereas the quartets were compared to the INH-RIF-PZA-EMB combination. Mice were administered only the dose that gives the human-equivalent exposure in mice for the individual drugs (where clinical data are available). In the case of SQ109 and BTZ043, the dose reported earlier to yield efficacy in the chronic mouse model was chosen for the combination study. Two of the 18 test combinations were not tolerated beyond the first week of dosing, and hence, these groups (MXF-THI-PA824 and MFX-THI-PA824-PZA) were terminated and excluded from the analysis. The reasons for their toxicity are not understood at this time. All the remaining triplet and quartet combinations were bactericidal compared to the results for the untreated controls (Fig. 2), thus translating successfully from *in vitro* bactericidal effect to *in vivo* bactericidal effect. Comparing the extent of bactericidal effect across the *in vitro* assays and the *in vivo* system for the triplet combinations, 5/8 regimens showed good correlation, while 3/8 regimens overperformed *in vitro* (Fig. 3). Among these combinations, EMB-BDQ-SQ109 and MXF-PA824-BDQ showed better efficacy (*P* < 0.01) than the reference 3-drug regimen (see Table S4 in the supplemental material).

All the triplet combinations gained further efficacy after the addition of PZA. We observed superior efficacy for five quartets compared to the INH-RIF-EMB-PZA combination (see Table S4 in the supplemental material).

## DISCUSSION

In a drug discovery cascade, the search for novel antimycobacterial leads often starts with a high-throughput screen with either the whole bacterium or a specific enzymatic target. The output of such a screen typically yields unoptimized chemical start points which are then advanced through various biological assays to derive a valuable clinical candidate. Such a strategy, while powerful for identifying individual lead compounds, is currently not amenable to identifying potentially valuable combinations that are central to the treatment of tuberculosis. We developed an *in silico* simulation platform that is equivalent to the above-described high-throughput screen and yields potentially useful combinations that can be advanced to further studies. Similar to a drug discovery cascade, the *in silico* output is only the start of a combination discovery cascade, wherein the subsequent progression depends on the discovery of bactericidal combinations *in vitro* and, finally, bactericidal combinations *in vivo*.

The *in silico* platform, which was developed in-house (and is not commercially available), describes the functioning of M. tuberculosis and its response to internal and external perturbations at the molecular and kinetic levels. The first such kinetic platform for E. coli was described previously (10). Based on a similar methodology, we have now constructed a dynamic platform for the pathogen M. tuberculosis. Such a framework responds to perturbations similarly to the way the natural system in question would respond. This type of modeling has been referred to as an “impossible” problem, primarily because of the dearth of parametric data required to give meaning to flux equations and secondarily because of the absence of robust software that can simulate and give stable solutions in systems comprising thousands of ordinary differential equations (ODEs). Our *in silico* platform has the ability to solve many thousands of simultaneous ODEs (10). The absence or the paucity of kinetic parameters could be overcome by numerically solving the ODEs and reverse engineering for the parameters that give stable solutions and are closest to the actual cellular physiology. In contrast, one of the major issues with ODE simulations is that we cannot generate a population data set because they yield single-cell output. Hence, we cannot model variability or heterogeneity. Second, our model simulates logarithmically growing mycobacteria. The various nongrowing (persistent/dormant bacteria) mycobacteria under alternate physiological states, such as altered oxygen levels, could not be simulated in our model.

None of the combinations in the entire set were antagonistic. Based on the pairwise validation observed (Fig. 1A), among the 816 triplets (18C3) that were studied in the *in silico* platform, we advanced only the top 50 triplet combinations from *in silico* to *in vitro* studies. The objective was to identify synergistic combinations that can be further advanced through *in vivo* studies, and hence, we focused on those combinations where the prediction was for a ∑FIC of <2 (i.e., synergistic and additive). However, the selection was not merely based on the best ΣFIC but also based on parameters such as adequate representation of all drug classes among the combinations and the ΣFIC in a given combination being a result of at least 10% of the MIC of each drug in the combination. The excellent correlation observed between *in silico* and *in vitro* results (Fig. 1B) corroborated our decision to advance such combinations to *in vitro* studies rather than take those from elsewhere in the rank order list that emerged from the *in silico* predictions.

The *in silico* model was optimized to predict growth and arrest of growth. Hence, it predicted the synergistic and additive relationship between drugs in the combination with respect to inhibition of growth, i.e., the best ΣFIC *in vitro*. However, the model could not predict bactericidal effect, because the current output parameters do not distinguish between arrest of growth and death. The assumption was that for a given combination to be bactericidal, it has to be inhibitory in the first place. Hence, our method of shortlisting based on growth inhibition was certainly more inclusive than exclusive in terms of identifying bactericidal combinations. The reasons that separated those combinations that were synergistic for bactericidal effect could not be discerned from the *in silico* platform. According to the work of Collins and colleagues, bactericidal effect in rapidly growing bacteria is due to the production of free radicals that follows a steep increase in the redox ratio (NAD/NADH) (51, 52). In our *in silico* E. coli platform, we could reproduce the increase in redox ratio with bactericidal targets (53). We observed experimentally that with bactericidal drugs, there is a significant production of free radicals in E. coli. However, in contrast, this increase is only marginal in the case of slowly growing M. tuberculosis (data not shown) and, therefore, does not serve as a suitable marker of bactericidal effect in the M. tuberculosis platform. Additionally, our attempt to correlate a fingerprint analysis of the flux of various metabolites with bactericidal effect was also unsuccessful. Thus, we have yet to identify a reliable metabolite signature of bactericidal effect in M. tuberculosis.

The choice of triplets for the *in vivo* efficacy studies from the list of bactericidal triplets was based on the criteria that all of the members were oral drugs and every drug was represented in at least one combination. Based on the bactericidal effect data (Table 2), regimens were selected in the median ± 0.5 log kill range (2.62 ± 0.5). From such a distribution, further bias was introduced such that the newer drugs, BDQ, PA824, and SQ109, which are in clinical trials, were adequately represented. The same was not possible with LZD, which is also in clinical trials, because with the exception of 1 combination, the remaining 6/7 combinations that had LZD yielded <2 log kill under *in vitro* conditions. A second study was planned to sample from the upper and the lower quartiles of the same distribution curve; however, due to the closure of the AstraZeneca India research and development site, where the *in vivo* studies would have been conducted, the subsequent set of *in vivo* studies was not possible.

The doses used in the *in vivo* studies were based on human-equivalent effective doses for each individual drug established under similar conditions. If viewed from the perspective of the *in vitro* FIC studies, then the drugs in a given combination were tested at 1 + 1 + 1 + 1 doses instead of the fractional doses which would have ideally tested the synergism observed from the *in silico* and *in vitro* studies. However, it is also pertinent to note that the relationship of the *in vitro*-derived FIC to plasma or tissue concentrations is poorly understood. Hence, the decision was to test full doses instead of fractional doses, in order to ensure maximum benefit with already approved human doses, which are known to minimize the risk of the emergence of resistance. Subsequent studies are needed to further titrate the doses to test whether the *in vitro*-derived FIC indices can serve as a guide to select doses *in vivo* in order to maximize the benefit of synergy while minimizing the risk of adverse events.

The standard triage of drug discovery involves potency testing *in vitro* (MIC studies), followed by efficacy testing in animal models after ensuring adequate plasma levels that merit the test of the hypothesis. In the present study, we employed a similar progression, wherein the combinations were first tested *in vitro* and then shortlisted for *in vivo* testing based on their effectiveness in killing M. tuberculosis. Among the nine triplets, CFZ-PA824-BDQ is advancing in a phase 2 trial (ClinicalTrials registration no. NCT01691534; 54). All of the BDQ-containing triplets (5/8) (Fig. 3, regimens 1, 2, 4, 6, and 7) and quartets (5/8) in our study were bactericidal; 5/5 quartets and 2/5 triplets among these were significantly superior, while the remaining 3 triplets were equivalent to the first-line regimen. Furthermore, 4/5 of these BDQ-containing triplets or quartets also contained PA824. The *in vitro* extent of kill correlated well with the *in vivo* extent of kill for all 5/5 triplets, thereby reinforcing the exquisite efficacy of BDQ and PA824 (56, 57). However, even among the triplets containing BDQ and PA824, there was a progressive increase in bactericidal effect observed depending on the inclusion of a more bactericidal agent (MFX > CFZ > EMB > SQ109). As an extension of the findings reported earlier by Reddy et al. (58), the regimen EMB-BDQ-SQ109 was significantly better than regimens in which EMB or SQ109 or CFZ was combined with PA824 and BDQ. Among the three triplets that overperformed in the *in vitro* studies (Fig. 3, regimens 3, 5, and 8), regimens 3 and 5 contained BTZ043, a compound which has been reported earlier (46) to be more potent *in vitro* than *in vivo*. The only oxazolidinone-containing regimen that was shortlisted for *in vivo* studies was inferior to the first-line regimen. This is in direct contrast to the other regimen containing EMB-SQ109, which has BDQ instead of LZD. Even under *in vitro* conditions, all the LZD-containing combinations performed moderately to poorly: 4/7 resulted in stasis, while 3/7 resulted in 1 to 2 Log_10_ CFU/ml kill rates (see Table S2 in the supplemental material). Only one among these was tested *in vivo*, and that was inferior to the first-line regimen. However, there are several lines of recent evidence to suggest the usefulness of oxazolidinone-containing regimens in patients (59, 60).

An often cited criticism in advancing compounds to *in vivo* studies based on *in vitro* bactericidal effect is the lack of its predictive value. This was partially reinforced from the outcome of these studies, where 3/8 test triplet combinations and the reference regimen either over- or underperformed under *in vitro* conditions (Fig. 3). The absence of a direct correlation can be explained by the following reasons: whereas under *in vitro* conditions, the bacilli are growing aerobically in a planktonic phase, under *in vivo* conditions in the mouse model, the bacilli are exclusively intracellular and in an apparently nonreplicating phase (61, 62). However, it is pertinent to note that in human lesions, all of these physiological states may be present during the course of the treatment. Hence, those combinations that are effective in both the *in vitro* and *in vivo* systems would be preferred. As a corollary, the *in silico* model described here pertains to only one of the physiological states and has significant room for further development. The limitation in predicting the usefulness of drugs like the oxazolidinone class is a caveat of our current *in silico* model. The model lacks the capability to discern metabolic improvements or any drug-drug interactions *in vivo*, as it is solely based on the mechanism of action. Moreover, the suitability of the modeling tool to simulate events following exposure to multidrug combinations that predict minimal relapse rates is as yet unexplored.

New versions of the *in silico* model would include the simulation of persistor bacteria, intracellular bacteria, etc., depending on the availability of biochemical knowledge of pathway behavior under these conditions. Recent studies exploring bactericidal effect under conditions of alternate carbon sources have revealed that there is accelerated killing under nonreplicating growth conditions when M. smegmatis is exposed to TMC207 in minimal medium containing glycerol as the carbon source, in contrast to a rich medium like LB (63). Our current model does not include metabolic pathways that are active when M. tuberculosis is grown in the presence of glycerol as the sole carbon source. Consequently, the *M. tuberculosis in silico* platform did not test the effect of drugs when carbon sources other than glucose are used. A few models that are used to study drug-drug interactions replicate events closer to *in vivo* conditions, and such systems have reported synergism or antagonism with respect to anti-TB drugs that is otherwise not evident from *in vitro* studies (64). All models, whether *in vitro*, *ex vivo*, or *in silico*, can only simulate the *in vivo* events to a certain extent. Furthermore, even *in vivo* animal models are incomplete with respect to modeling human disease in its entirety, with the exception of nonhuman primate studies. However, the outcomes are sometimes unexplainable, perhaps due to the underlying mechanisms of action of these drugs, which can vary based on the environment (e.g., PA824 under hypoxic versus aerobic conditions [37, 65]), or simply due to the occurrence of complex and variable changes occurring within the host as part of disease progression. The prowess of the *in silico* approach described here is that the computational architecture and power can be extended to study any physiological state in a high-throughput mode, provided the biochemistry of pathways under such physiological conditions is even partially understood. The current study did not evaluate nonbeneficial combinations *in vitro* and *in vivo*, and hence, the negative predictive value of the *in silico* platform model is unknown. However, through our modeling and subsequent experimental methods, we have sifted through a large number of potentially useful combinations and unraveled at least five that are significantly better than the standard regimen containing INH, RIF, EMB, and PZA, thus paving the way for better options in the care and management of tuberculosis patients.

## Supplementary Material

Supplemental material
