# Supplementary material for: In Silico-Based High-Throughput Screen for Discovery of Novel Combinations for Tuberculosis Treatment
Source: Antimicrob Agents Chemother. 2015 Aug 14;59(9):5664–74. doi: 10.1128/AAC.05148-14 (PMC4538536; doi:10.1128/AAC.05148-14)
Supplement: Supplemental material [file supp_59_9_5664__index.html]

 In Silico-Based High-Throughput Screen for Discovery of Novel Combinations for Tuberculosis Treatment — Supplemental material 

# *In Silico*-Based High-Throughput Screen for Discovery of Novel Combinations for Tuberculosis Treatment

## Supplemental material

- Supplemental file 1 -

  Supplemental information on drug mechanism incorporation in the *in silico* platform, Figures S1 to S4, and Tables S1 to S4.

  PDF, 1.8M
