# Supplementary material for: In Silico-Based High-Throughput Screen for Discovery of Novel Combinations for Tuberculosis Treatment
Source: Antimicrob Agents Chemother. 2015 Aug 14;59(9):5664–74. doi: 10.1128/AAC.05148-14 (PMC4538536; doi:10.1128/AAC.05148-14)
Supplement: Supplemental material [file AAC.05148-14_zac009154347so1.pdf]

Title: *In silico* based high through put screen for discovery of novel combinations for the treatment for tuberculosis

Authors: Ragini Singh[1*], Vasanthi Ramachandran[2*‡], Radha Shandil[2*†], Sreevalli Sharma[2‡], Swati Khandelwal[1], Malancha Karmarkar[1‡], Naveen Kumar[2], Suresh Solapure[2†], RamanathaSaralaya[2], Robert Nanduri[2], Vijender Panduga[2], Jitendar Reddy[2], K. R. Prabhakar[2], Swaminathan Rajagopalan[1], Narasimha Rao[1], Shridhar Narayanan[2†], Anand Anandkumar[1‡], V. Balasubramanian[1#‡], Santanu Datta[1‡]

Affiliations:

[1]Cellworks Research India Pvt. Ltd., #118, 3rd floor, West Wing, Neil-Rao Towers, Road No: 3, EPIP, Bengaluru 560066, India.

[2]AstraZeneca India Pvt. Ltd., Kirloskar Business Park, Bellary Road, Hebbal, Bengaluru 560024, India.

#Correspondence to: bala.subramanian@cellworksgroup.com; Phone: +919845398063

*Joint first authors; Ragini Singh led the *in silico* modelling, Vasanthi Ramachandran led the *in vitro* studies and Radha Shandil led the *in vivo* studies.

[†]Present address: Foundation for Neglected Disease Research, NCBS Campus, Bellary Road, Bengaluru 560065, India.

[‡]Present Address: Bugworks Research India Pvt. Ltd. Suite 105, EVOMA, 88 Borewell Road, Whitefield, Bengaluru 560066, India

Running Head: *In silico* derived drug combinations for tuberculosis

Table of Contents

1. Drug mechanism incorporation in the *in silico* platform
2. **Fig. S1**: Validation of the *in silico* platform using *in vitro* 2-D checkerboard analysis. Representative data set depicting the synergy between RIF and SQ109. (A) Outcome from the *in silico* platform. (B) Isobologram from the *in vitro* checkerboard experiment.
3. **Fig. S2:** Inter-day reproducibility in the 2-D *in vitro* checkerboard assays. Panels A-F are isobolograms of representative pairs of drugs. Panel G is correlation of the best ∑FIC for each pair tested across two experiments.
4. **Fig. S3:** Synergy between CFZ, PA824, BDQ. Squares (*in silico*) and Circles (*in vitro*) are the (x,y,z) coordinates pertaining to the FIC indices of the three drugs where inhibition of *M. tuberculosis* growth was observed.
5. **Fig. S4:** Bactericidality observed in the 3-drug combination: CFZ (C), PA824 (P), BDQ (T). Numbers in parenthesis indicate the FIC at which the drug was tested.
6. **Table S1:** Data from 51 Triplets; *in silico* FIC Analysis where the ΣFIC for each triplet across the various concentrations where Biomass arrest was observed in the platform.
7. **Table S2:** *In vitro* ∑FIC and bacterial reduction (ΔLog_{10}CFU/ml) for each triplet.
8. **Table S3:** Steady state exposure (AUC) and Peak concentration (Cmax) observed for a drug in the 3- and 4-drug combination.
9. **Table S4:** One way ANOVA with Dunnett's Multiple Comparisons Test. P values marked with asterix are groups which are superior to the reference combination.

DRUG MECHANISM INCORPORATION IN THE IN SILICO PLATFORM

Abbreviation index:

Vf = Velocity of the flux

Km = Michaelis-Menten constant; the substrate's affinity towards the enzyme

Vf_app = Apparent Velocity due to action of an inhibitor/activator

Km_app = Apparent Michaelis-Menten constant due to action of an inhibitor/activator.

VCyt = Volume of the cytoplasm

kcatf = turnover number for the forward flux

kcatr = turnover number for the backward flux

1. ISONIAZID (*11-14*)

TARGET: Enoyl-ACP Reductase (inh A)

INHIBITION TYPE: Competitive inhibition, following covalent addition to co-factor

MOA: Catalase peroxidase, KatG activates the pro-drug Isoniazid. The KatG reacts with both NADH and INH. NADH is converted to NAD+ which replaces the hydrazine moiety of the anti-tubercular pro-drug INH to generate the active form of the drug, isonicotinoyl-NAD. This isonicotinoyl-NAD binds to the co-factor binding site of InhA, i.e. the NADH binding site. It has been seen that this is a two-step inhibition mechanism wherein initial enzyme–inhibitor complex (EI) and the final enzyme–inhibitor complex (EI*), is formed. It has been seen that the INH--NAD is a slow tight binding reversible inhibitor that interacts rapidly with the enzyme to form an initial complex, EI, that then slowly converts to a more stable (i.e. more strongly bound) final inhibited complex, EI*.

EQUATION: INH Pro-drug + NADH or NADPH = 4S-isomer INH-NAD adduct + Other INH-NAD & INH-NADP adducts. Only 4S-isomer INH-NAD adduct binds to InhA, enoyl ACP-reductase. Concentration of effective INH-NAD adduct, the 4S-Isomer is reduced to 1/12th of original MIC.

2. RIFAMPICIN (*15*)

TARGET: *rpoB*, β subunit of bacterial RNA polymerase (RNAP),; Transcription

INHIBITION TYPE: Non-competitive inhibition

MOA: Rifampicin blocks the transcript passage way, thus preventing the translation process to commence on the nascent mRNA. So RIF serves as a steric block. The equation is also written on the same basis of non-competitive inhibition because the RNAP is stalled and can no longer perform its function. Its activity is affected there the inhibition shown on Vmax.

EQUATION: (Vf_MTB_RNAP_app/(([MTB_topA_psu]/Ka_MTB_topA_psu)*([MTB_dnaB_psu]/Ka_MTB_dnaB_psu)*([MTB_gyrAB_psu]/Ka_MTB_gyrAB_psu))*[MTB_A_transcription])/ (Km_MTB_A_transcription+[MTB_A_transcription]))

Vf_MTB_RNAP_app = Vf_MTB_RNAP/(1+([Rifampicin]/Ki_Rifampicin))

3. ETHAMBUTOL (*16-18*)

TARGET: Arabinosyltransferase

INHIBITION TYPE: Non-competitive inhibition

MOA: Ethambutol is a synthetic amino alcohol (ethylene diaminodi1butanol). Primary site of action is arabinan synthesis in both the structural polysaccharide arabinogalactan and the wall-associatedlipoglycan, lipoarabinomannan. It hinders the arabinosylation which is catalyzed by arabinosyltransferases embA, embB for AG and embC for LAM. EmbR is the transcriptional regulator of the polycistronic operon embCAB. EmbR is a multidomain protein with a forkhead-associated domain, FHA, which s a phosphoprotein recognition domain. This domain was found to play a crucial role in the interaction and phosphorylation of EmbR by PknH, a serine/threonine protein kinase (STPK) of *M. tuberculosis.* The inhibition is shown on all the enzymes: embA, embB and embC.

EQUATIONS:

1.((Vf_MTB_embA_embB_aftC_app*[MTB_Decaprenyl_p_fura_d_Arabinose])*[MTB_Decaprenyl_p_GlcNAc_p_Rha_fura_d_Galactose_30_fura_d_Arabinose_3])/((((Kd_MTB_Decaprenyl_p_fura_d_Arabinose*Km_MTB_Decaprenyl_p_GlcNAc_p_Rha_fura_d_Galactose_30_fura_d_Arabinose_3)+(Km_MTB_Decaprenyl_p_GlcNAc_p_Rha_fura_d_Galactose_30_fura_d_Arabinose_3*[MTB_Decaprenyl_p_fura_d_Arabinose]))+(Km_MTB_Decaprenyl_p_fura_d_Arabinose*[MTB_Decaprenyl_p_GlcNAc_p_Rha_fura_d_Galactose_30_fura_d_Arabinose_3]))+([MTB_Decaprenyl_p_fura_d_Arabinose]*[MTB_Decaprenyl_p_GlcNAc_p_Rha_fura_d_Galactose_30_fura_d_Arabinose_3]))

Vf_MTB_embA_embB_aftC_app = Vf_MTB_embA_embB_aftC / (1+([Ethambutol] /Ki_Ethambutol))

2.((Vf_MTB_embC_app*[MTB_Decaprenyl_p_fura_d_Arabinose])*[MTB_LM_IM3])/(((Km_MTB_LM_IM3*[MTB_Decaprenyl_p_fura_d_Arabinose])+(Km_MTB_Decaprenyl_p_fura_d_Arabinose*[MTB_LM_IM3]))+([MTB_Decaprenyl_p_fura_d_Arabinose]*[MTB_LM_IM3]))

3.((Vf_MTB_embC_app*[MTB_Decaprenyl_p_fura_d_Arabinose])*[MTB_LM])/(((Km_MTB_LM*[MTB_Decaprenyl_p_fura_d_Arabinose])+(Km_MTB_Decaprenyl_p_fura_d_Arabinose*[MTB_LM]))+([MTB_Decaprenyl_p_fura_d_Arabinose]*[MTB_LM]))

Vf_MTB_ embC_app = Vf_MTB_embC / (1+([Ethambutol] /Ki_Ethambutol))

4. AMIKACIN (*19*)

TARGET: Translation Machinery; 30S Ribosome

INHIBITION TYPE: Noncompetitive inhibition

MOA: In case of binding of a non-cognate tRNA to the A-site there is a conformational change induced in the 30S ribosomal subunit. When in normal state it identifies the non-cognate tRNA and modifies it for proper functional protein synthesis. When bound to amikacin or kanamycin it cannot overcome the non-cognate RNA being incorporated and leads to dysfunctional protein synthesis. Therefore aminoglycosides changes the Kf i.e. rate of the forward reaction is affected.

EQUATION: (on the 30S formation subunit)

(Vf_app*[MTB_30S_Source])/(Km_MTB_30S_Source+[MTB_30S_Source])

Vf_app= Vf/(1+([Amikacin]/Ki_Amikacin))

5. STREPTOMYCIN (*20*)

TARGET: Translation machinery, 30S Ribosome

INHIBITION TYPE: Non-competitive inhibition

MOA: The 30S subunit is usually involved with the high fidelity of translation. If this undergoes a conformational change it leads to mis-incorporation of amino acids which leads to dysfunctional protein formation finally leading to cell death. When Streptomycin binds to 30S it leads to a decreased fidelity conformational state referred to as “ram” or ribosomal ambiguity. So the processivity is reduced and thereby the drug acts on the Vmax serving as a non-competitve inhibitor.

EQUATION: (on the 30S formation subunit)

((Vf_app*[MTB_30S_Source])/(Km_MTB_30S_Source+[MTB_30S_Source])

Vf_app = Vf/(1+([Streptomycin]/Ki_Streptomycin))

6. KANAMYCIN (*21,22*)

TARGET: Translation Machinery; 30S Ribosome

INHIBITION TYPE: Noncompetitive inhibition

MOA: In case of binding of a non-cognate tRNA to the A-site there is a conformational change induced in the 30S ribosomal subunit. When in normal state it identifies the non-cognate tRNA and modifies it for proper functional protein synthesis. When bound to amikacin or kanamycin it cannot overcome the non-cognate RNA being incorporated and leads to dysfunctional protein synthesis. Therefore aminoglycosides changes the Kf i.e. rate of the forward reaction is affected.

EQUATION: (on the 30S formation subunit)

((Vf_app*[MTB_30S_Source])/(Km_MTB_30S_Source+[MTB_30S_Source])

Vf_app= Vf/(1+([Kanamycin]/Ki_Kanamycin))

7. CAPREOMYCIN (*23,24*)

TARGET: 16S-23S rRNA; Translation Machinery

INHIBITION TYPE: Non-competitive inhibition

MOA: Capreomycin is bound to a cleft formed by 16S rRNA binding to 23S rRNA subunits. This in turn stabilizes the tRNA in the in the A-site in the pre-translocation state. Hence the forward rate of the translation process is affected. So the reaction is written as Kf_app.

EQUATION: (on the 50S formation subunit)

(Vf_app*[MTB_50S_Source])/(Km_MTB_50S_Source+[MTB_50S_Source])

Vf_app = (Vf/(1+([Capreomycin]/Ki_Capreomycin))

8. CLARITHROMYCIN (*25*)

TARGET: Translation Machinery

INHIBITION TYPE: Non-Competitive inhibition

MOA: Clarithromycin binds to the 50S and inhibits its activity leading to inhibition of transpeptidation, translocation, chain elongation and, ultimately, bacterial protein synthesis.

EQUATION: (on the 50S formation subunit)

(Vf_app*[MTB_50S_Source])/(Km_MTB_50S_Source+[MTB_50S_Source])

Vf_app = Vf / (1+([Clarothromycin]/Ki_Clarithromycin))

9. MOXIFLOXACIN (*26,27*)

TARGET: Replication Machinery

INHIBITION TYPE: Non-Competitive inhibition

MOA: The drug is depicted as non-competitive inhibitor because it brings about a conformational change which stalls the activity of the gyrase complex.

EQUATION:

(Vf_app*[MTB_gyrA_Source])/(Km_MTB_gyrA_Source+[MTB_gyrA_Source])

Vf_app = Vf/(1+([Moxifloxacin]/Ki_Moxifloxacin))

10. MEROPENEM (*28,29*)

TARGET: Cell Wall Inhibition

INHIBITION TYPE: Competitive inhibition

MOA: Meropenem is an irreversible covalent inhibitor of PBP. It binds competitively to the substrate.

EQUATIONS:

1. 1. (Vf_MTB_pbpB*[MTB_UNAM_NAG]) / (Km_MTB_UNAM_NAG_app+ [MTB_UNAM_NAG])

Km_MTB_UNAM_NAG_app= Km_MTB_UNAM_NAG*(1+[(Meropenem]/Ki_Meropenem))

2. 2. (Vf_MTB_pbpB*[MTB_DNAM_NAG]) / (Km_MTB_DNAM_NAG_app+[MTB_DNAM_NAG])

Km_MTB_DNAM_NAG_app= Km_MTB_DNAM_NAG*(1+([Meropenem]/Ki_Meropenem)

3. 3. (Vf_MTB_pbpB*[MTB_PG_LAYERS]) / (Km_MTB_PG_LAYERS_app+[MTB_PG_LAYERS])
Km_MTB_PG_LAYERS_app= Km_MTB_PG_LAYERS*(1+([Meropenem]/Ki_Meropenem))

11. D-CYCLOSERINE (*30,31*)

TARGET: Cell wall biosynthesis

INHIBITION TYPE: Competitive inhibition

MOA: DCS is a cyclic structural analog of D-alanine. The drug interferes with the activities of both the enzymesD-alanine racemase (**Alr**) and D-alanine:D-alanine ligase (**Ddl**). . It binds to the active site of these enzymes because their substrate is D-ala. So it serves as a competitive inhibitor for both the enzymes and prevents peptidoglycan synthesis. Peptidoglycan layer in bacterial cell wall is required for resistance towards osmotic pressure.

EQUATIONS:

1. (((((kcatf_alr*Km_DALA)*[MTB_LALA]) ((kcatr_alr*Km_LALA_app)*[MTB_DALA]))*[MTB_alr])*VCyt)/(((Km_LALA_app*[MTB_DALA])+(Km_DALA*[MTB_LALA]))+ (Km_LALA_app*Km_DALA))

Km_LALA_app = Km_LALA*(1+([MTB_DCS]/Ki_DCS))

2. (Vf*([MTB_ATP]*[MTB_DALA]))/((((Kd_ATP*Km_DALA_app)+ (Km_DALA_app*[MTB_ATP]))+(Km_ATP*[MTB_DALA]))+ ([MTB_ATP]*[MTB_DALA]))
Km_DALA_app = Km_DALA*(1+([MTB_DCS]/Ki_DCS))

12. CLOFAZIMINE (*32*)

TARGET: Respiratory cycle

INHIBITION TYPE: Competitive inhibition

MOA: The primary respiratory chain NADH:quinone oxidoreductase (NDH-2) is a membrane bound protein of 50 kDa containing a single FAD moiety. It typically catalyzes the transfer of electrons from NADH to menaquinone, the only quinone type in mycobacteria, thereby contributing to respiratory chain activity in mycobacteria. Menaquinol continues the transfer by supplying electrons/H+ to downstream respiratory chain oxidoreductases, which in turn use the electrons/ H+ to reduce O2. Clofazimine is reduced by the oxidoreductase NDH2. Direct transfer of electrons/H+ from NADH to clofazimine has been reported. Clofazimine is unstable and spontaneously reacts with O2 to produce reactive oxygen species (ROS). Therefore there is a cyclical production of ROS that is fueled by NADH and O2. This cycle will continually run because NADH is a key substrate in generating ATP and is continually produced by the citric acid cycle and/or betaoxidation of fatty acids.

Clofazimine is not an inhibitor but a second substrate for the enzyme NDH2. Hence when clofazimine binds to NDH2 it leads to reduction of clofazimine followed by ROS production and cell death. So inhibition is achieved by binding of a second substrate to the same site yielding toxic product leading to cell death.

EQUATIONS:

1. ((Vf_MTB_ndh*[MTB_NADH])*[MTB_UQ])/(((((a*Km_MTB_NADH)*Km_MTB_UQ_app)+((a*Km_MTB_NADH)*[MTB_UQ]))+((a*Km_MTB_UQ_app)*[MTB_NADH]))+([MTB_NADH]*[MTB_UQ]))

2. ((Vf_MTB_nuo*[MTB_NADH])*[MTB_UQ])/(((((a*Km_MTB_NADH)*Km_MTB_UQ_app)+ ((a*Km_MTB_NADH)*[MTB_UQ]))+((a*Km_MTB_UQ_app)*[MTB_NADH]))+([MTB_NADH]*[MTB_UQ]))Km_MTB_UQ_app = Km_MTB_UQ*((1+([Clofazimine]/Ki_Clofazimine))

13. THIACETAZONE (*33*)

TARGET: Cell wall biosynthesis

INHIBITION TYPE: Non-Competitive inhibition

MOA: After the elongation of fatty acid by FAS II, meromycolates fail to be cyclopropanated when thiacetazone (TAC) is added. Inhibition causes reduction of enzyme activity leading to substrate accumulation. EthA is a NADPH specific FAD containing monooxygenase which is responsible for TAC activation.

EQUATIONS

1. ((Vf_mmaA2_app*[MTB_S_adenosyl_met])*[MTB_cis_met_hydroxymethyl_C56_acylACP])/((((Kd_S_adenosyl_met*Km_cis_met_hydroxymethyl_C56_acylACP)+(Km_cis_met_hydroxymethyl_C56_acylACP*[MTB_S_adenosyl_met]))+(Km_S_adenosyl_met*[MTB_cis_met_hydroxymethyl_C56_acylACP]))+([MTB_S_adenosyl_met]*[MTB_cis_met_hydroxymethyl_C56_acylACP]))

    Vf_mmaA2_app = Vf_mmaA2/(1+([TAC]/Ki_TAC))

2. ((Vf_cmaA2_app*[MTB_S_adenosyl_met])*[MTB_trans_methylketo_C60_acylACP])/((((Kd_S_adenosyl_met*Km_trans_methylketo_C60_acylACP)+(Km_trans_methylketo_C60_acylACP*[MTB_S_adenosyl_met]))+(Km_S_adenosyl_met*[MTB_trans_methylketo_C60_acylACP]))+([MTB_S_adenosyl_met]*[MTB_trans_methylketo_C60_acylACP]))

    Vf_cmaA2_app = Vf_cmaA2/(1+([TAC]/Ki_TAC))

3. ((Vf_pcaA_app*[MTB_S_adenosyl_met])*[MTB_cis_enoyl_cp_C53_acylACP])/((((Kd_S_adenosyl_met*Km_cis_enoyl_cp_C53_acylACP)+(Km_cis_enoyl_cp_C53_acylACP*[MTB_S_adenosyl_met]))+(Km_S_adenosyl_met*[MTB_cis_enoyl_cp_C53_acylACP]))+([MTB_S_adenosyl_met]*[MTB_cis_enoyl_cp_C53_acylACP]))

Vf_pcaA_app = Vf_pcaA/(1+([TAC]/Ki_TAC))

14. BEDAQUILINE (BDQ; formerly known as TMC207; *34,35*)

TARGET: ATP Synthase; Energy generation

INHIBITION TYPE: Competitive inhibition

MOA: The drug competes with the Hydrogen ion and blocks ATP synthase. Bedaquiline binds to the subunit c of the membrane embedded F0 sector. Protons binds to an essential acidic residue of subunit c. So it is a competitive inhibition by the drug. The acidic residue is Glu61.

But due to modeling constraints, this particular enzyme mediated equation was remodeled as a Hill equation and inhibition shown to be non-competitive. The drug affects the Vf_MTB_bo_app.

EQUATION:

((Vf_MTB_bo_app*[MTB_UQH2])*[MTB_ADP]^1.5)/(((((a*Km_MTB_UQH2)*Km_MTB_ADP^1.5)+ ((a*Km_MTB_UQH2)*[MTB_ADP]^1.5))+((a*Km_MTB_ADP^1.5)*[MTB_UQH2]))+ ([MTB_UQH2]*[MTB_ADP]^1.5))

Vf_MTB_bo_app= Vf_MTB_bo / (1+([BDQ]/Ki_BDQ))

15. LINEZOLID (*36*)

TARGET: Translation Machinery

INHIBITION TYPE: Non-Competitive inhibition

MOA: Linezolid binds to the 23S rRNA of the 50S subunit of ribosome. It binds to the A-site portion of the peptidyl tranferase centre overlapping the site of the aminoacyl moiety of A-site bound tRNA. The linezolid binding pocket is lined by eight 23S rRNA nucleotides that are highly conserved among the three domains of life. Upon binding there is a subtle conformational change. In the model the 70S is shown to be formed from the binding of 30S and 50S subunits. So if there is an inhibition on the 50S subunit the ribosomal complex is not formed.

EQUATION: (on 50S formation subunit)

(Vf_app*[MTB_50S_Source])/(Km_MTB_50S_Source+[MTB_50S_Source])

Vf_app = Vf / (1+([Linezolid]/Ki_Linezolid)

16. PA824 (*37*)

TARGET: Energy metabolism and Cell Wall Inhibition

INHIBITION TYPE: Mixed

MOA: PA824 is a prodrug reductively activated by a deazaflavin, F(420), dependent nitroreductase (Ddn). Treatment of aerobically replicating cells with PA824 rapidly disrupts the formation of ketomycolates with concomitant accumulation of hydroxymycolates, a class of mycolic acids that are major constituents of the cell envelope of *M. tuberculosis*. PA824 has a complex mode of action with significant effects on transcription of genes responsive to known inhibitors of cell wall synthesis (such as isoniazid, thiolactomycin, ethionamide and cerulenin) as well as on genes responsive to respiratory poisons (such as potassium cyanide).

EQUATIONS:

a) ***Noncompetitive inhibition on cell wall targets:***

1. ((Vf_MTB_mmaA1_app*[MTB_S_adenosyl_l_methionine])*[MTB_cis_delta_2_enoyl_C54_acyl_ACP])/((((Kd_MTB_S_adenosyl_l_methionine*Km_MTB_cis_delta_2_enoyl_C54_acyl_ACP)+(Km_MTB_cis_delta_2_enoyl_C54_acyl_ACP*[MTB_S_adenosyl_l_methionine]))+(Km_MTB_S_adenosyl_l_methionine*[MTB_cis_delta_2_enoyl_C54_acyl_ACP]))+([MTB_S_adenosyl_l_methionine]*[MTB_cis_delta_2_enoyl_C54_acyl_ACP]))

2. ((Vf_MTB_mmaA1_app*[MTB_S_adenosyl_l_methionine])*[MTB_cis_met_hydroxy_C59_acyl_ACP])/((((Kd_MTB_S_adenosyl_l_methionine*Km_MTB_cis_met_hydroxy_C59_acyl_ACP)+(Km_MTB_cis_met_hydroxy_C59_acyl_ACP*[MTB_S_adenosyl_l_methionine]))+(Km_MTB_S_adenosyl_l_methionine*[MTB_cis_met_hydroxy_C59_acyl_ACP]))+([MTB_S_adenosyl_l_methionine]*[MTB_cis_met_hydroxy_C59_acyl_ACP]))

3. ((Vf_MTB_mmaA3_app*[MTB_S_adenosyl_l_methionine])*[MTB_cis_met_hydroxy_C59_acyl_ACP])/((((Kd_MTB_S_adenosyl_l_methionine*Km_MTB_cis_met_hydroxy_C59_acyl_ACP)+(Km_MTB_cis_met_hydroxy_C59_acyl_ACP*[MTB_S_adenosyl_l_methionine]))+(Km_MTB_S_adenosyl_l_methionine*[MTB_cis_met_hydroxy_C59_acyl_ACP]))+([MTB_S_adenosyl_l_methionine]*[MTB_cis_met_hydroxy_C59_acyl_ACP]))

4. (Vf_MTB_mmaA3_app*[MTB_S_adenosyl_l_methionine])*[MTB_trans_met_hydroxy_C60_acyl_ACP])/((((Kd_MTB_S_adenosyl_l_methionine*Km_MTB_trans_met_hydroxy_C6

0_acyl_ACP)+(Km_MTB_trans_met_hydroxy_C60_acyl_ACP*[MTB_S_adenosyl_l_methionine]))+ (Km_MTB_S_adenosyl_l_methionine*[MTB_trans_met_hydroxy_C60_acyl_ACP]))+ ([MTB_S_adenosyl_l_methionine]*[MTB_trans_met_hydroxy_C60_acyl_ACP]))

5. ((Vf_MTB_mmaA3_app*[MTB_S_adenosyl_l_methionine])*[MTB_cis_met_hydroxy_C55_acyl_ACP])/((((Kd_MTB_S_adenosyl_l_methionine*Km_MTB_cis_met_hydroxy_C55_acyl_ACP)+(Km_MTB_cis_met_hydroxy_C55_acyl_ACP*[MTB_S_adenosyl_l_methionine]))+ (Km_MTB_S_adenosyl_l_methionine*[MTB_cis_met_hydroxy_C55_acyl_ACP]))+ ([MTB_S_adenosyl_l_methionine]*[MTB_cis_met_hydroxy_C55_acyl_ACP]))

6. ((Vf_MTB_mmaA3_app*[MTB_S_adenosyl_l_methionine])*[MTB_trans_met_hydroxy_C56_acyl_ACP])/((((Kd_MTB_S_adenosyl_l_methionine*Km_MTB_trans_met_hydroxy_C56_acyl_ACP)+(Km_MTB_trans_met_hydroxy_C56_acyl_ACP*[MTB_S_adenosyl_l_methionine]))+ (Km_MTB_S_adenosyl_l_methionine*[MTB_trans_met_hydroxy_C56_acyl_ACP]))+ ([MTB_S_adenosyl_l_methionine]*[MTB_trans_met_hydroxy_C56_acyl_ACP]))

Vf_MTB_mmaA1_app = Vf_MTB_mmaA1/(1+([PA824]/Ki_PA824))

Vf_MTB_mmaA3_app = Vf_MTB_mmaA3/(1+([PA824]/Ki_PA824))

b) Competitve inhibition for the respiratory target:

7. ((Vf_MTB_bo*[MTB_UQH2])*[MTB_ADP]^1.5)/

(((((a*Km_MTB_UQH2_app)*Km_MTB_ADP^1.5)+ ((a*Km_MTB_UQH2_app)*[MTB_ADP]^1.5))+ ((a*Km_MTB_ADP^1.5)*[MTB_UQH2]))+ ([MTB_UQH2]*[MTB_ADP]^1.5))
Km_MTB_UQH2_app = Km_MTB_UQH2 * (1+([PA824]/Ki_PA824))

17. SQ109 (*38*)

TARGET: Cell Wall Inhibition

INHIBITION TYPE: Non-Competitive inhibition

MOA: The mode of action remains unclear. SQ109 a 1,2-diamine related to ethambutol interferes with the assembly of mycolic acids into the cell wall core of *M. tuberculosis*, as bacilli exposed to SQ109 show immediate inhibition of trehalosedimycolate (TDM) production and fail to attach mycolates to the cell wall arabinogalactan. MmpL3, the target of SQ109 is a transporter of the mycobacterial trehalose mono mycolate, TMM. The drug interferes with the functionality of the enzyme MmpL3. So it has been shown to inhibit noncompetitively.

EQUATIONS:

1. ((Vf_MTB_MmpL3_app*[MTB_alpha_Trehalose_MM])*[MTB_ATP])/ ((Km_MTB_alpha_Trehalose_MM+[MTB_alpha_Trehalose_MM])*(Km_MTB_ATP+ [MTB_ATP]))

2. ((Vf_MTB_MmpL3_app*[MTB_cis_methoxy_Trehalose_MM]) *[MTB_ATP])/((Km_MTB_cis_methoxy_Trehalose_MM+ [MTB_cis_methoxy_Trehalose_MM])* (Km_MTB_ATP+[MTB_ATP]))

3. ((Vf_MTB_MmpL3_app*[MTB_trans_methoxy_Trehalose_MM]) *[MTB_ATP])/((Km_MTB_trans_methoxy_Trehalose_MM+ [MTB_trans_methoxy_Trehalose_MM]) *(Km_MTB_ATP+[MTB_ATP]))

4.((Vf_MTB_MmpL3_app*[MTB_cis_keto_Trehalose_MM]) *[MTB_ATP])/((Km_MTB_cis_keto_Trehalose_MM +[MTB_cis_keto_Trehalose_MM])*(Km_MTB_ATP+[MTB_ATP]))

5.((Vf_MTB_MmpL3_app*[MTB_trans_keto_Trehalose_MM]) *[MTB_ATP])/((Km_MTB_trans_keto_Trehalose_MM +[MTB_trans_keto_Trehalose_MM]) * (Km_MTB_ATP+[MTB_ATP]))

Vf_MTB_MmpL3_app = Vf_MTB_MmpL3 /(1+([SQ109]/Ki_SQ109))

18. BTZ043 (*39*)

TARGET: Cell wall Biosynthesis

INHIBITION TYPE: Non-Competitive inhibition

MOA: BTZ043 is an electron deficient nitro-aromatic compound which when reduced in the biological system yields nitrosoarene. This is nitrosoderivative of BTZ reacts with the thiol group of the enzyme DprE1 and forms a semi mercaptal adduct of activated BTZ and DprE1. Because of the covalent modification it interferes with the activity of the enzyme i.e. it does not allow the conversion of decaprenylphosphoryl-β-D-ribofuranose (DPR) into decaprenylphosphoryl-β-D-arabinose (DPA)

EQUATIONS:

(((((kcatf_DprE1_app*Km_DPKEF)*[MTB_DPR]) ((kcatr_DprE1*Km_DPR)*[MTB_DPKEF]))*[MTB_dprE1])*VCyt)/

(((Km_DPR*[MTB_DPKEF])+(Km_DPKEF*[MTB_DPR]))+ (Km_DPR*Km_DPKEF)) kcatf_DprE1_app = kcatf_DprE1/(1+([BTZ]/ Ki_BTZ))

Fig. S1: Validation of the *in silico* platform using in vitro 2-D checkerboard analysis. Representative data set depicting the synergy between RIF and SQ109. (A) Outcome from the *in silico* model. (B) Isobologram from the *in vitro* checkerboard experiment.

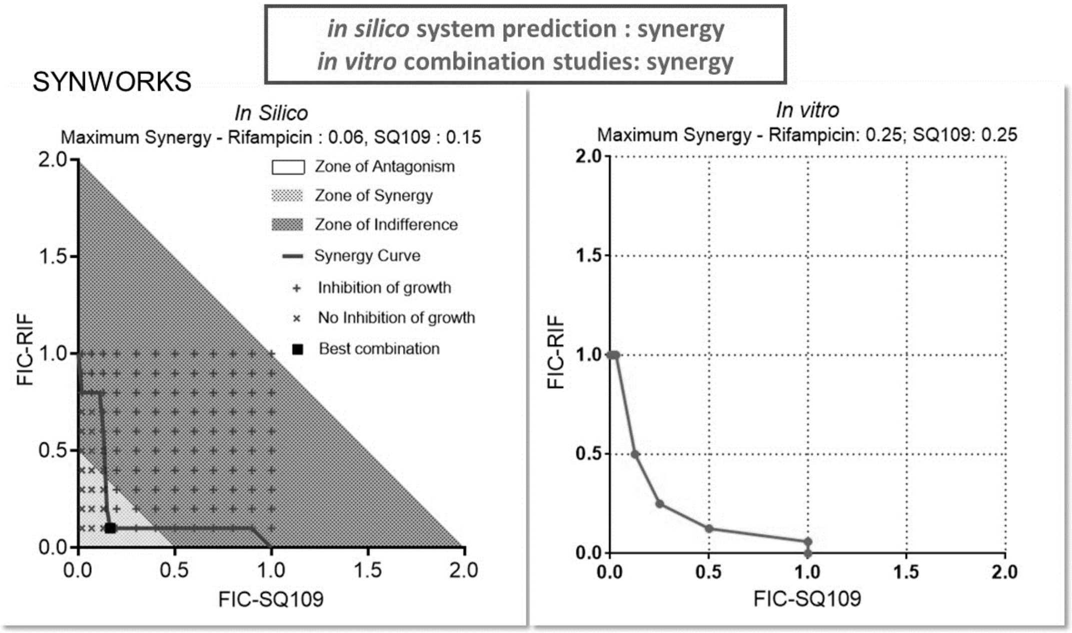

Fig. S2: Inter-day reproducibility in the 2-D in vitro checkerboard assays. Panels A-F are isobolograms of representative pairs of drugs. Panel G is correlation of the best ∑FIC for each pair tested across two experiments.

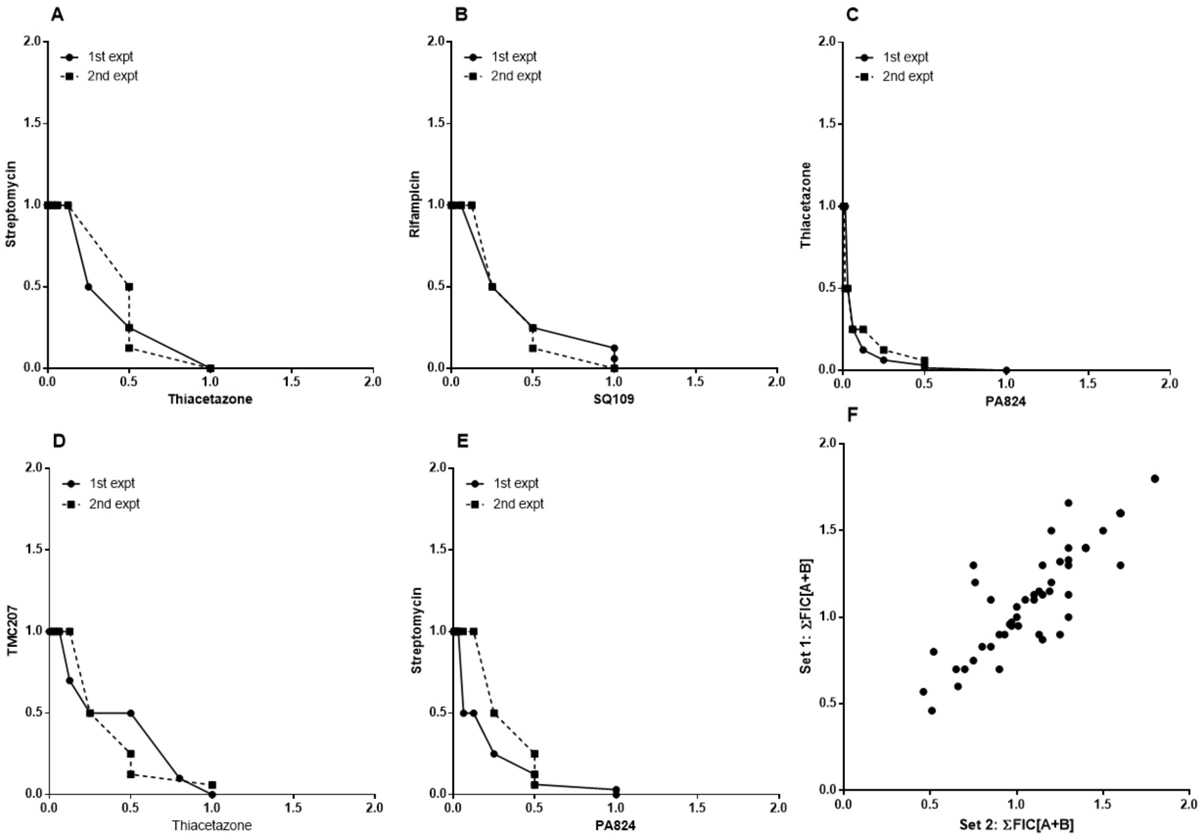

Fig. S3: Synergy between Clofazimine, PA824, Bedaquiline. Squares (in silico) and Circles (in vitro) are the (x,y,z) coordinates pertaining to the FIC indices of the three drugs where inhibition of *M. tuberculosis* growth was observed.

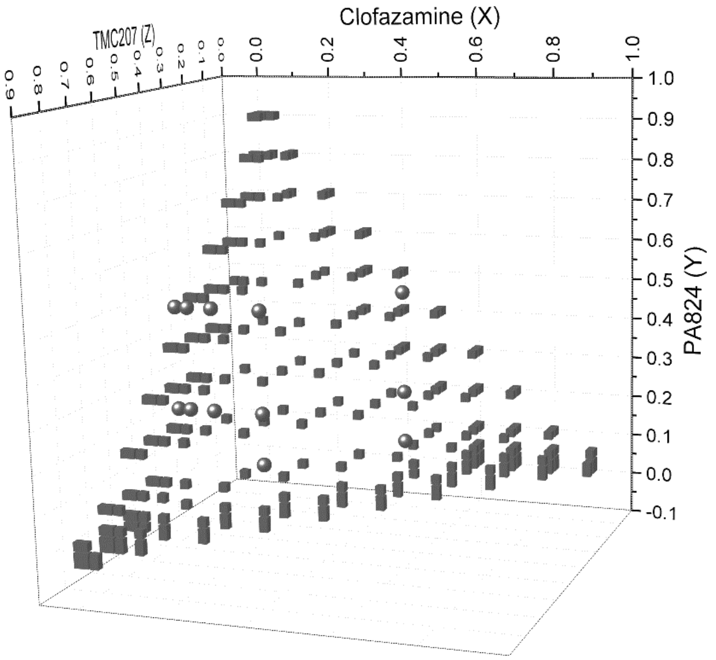

Fig. S4: Bactericidality observed in the 3-drug combination: Clofazimine (C), PA824 (P), Bedaquiline (T). Numbers in parenthesis indicate the FIC at which the drug was tested.

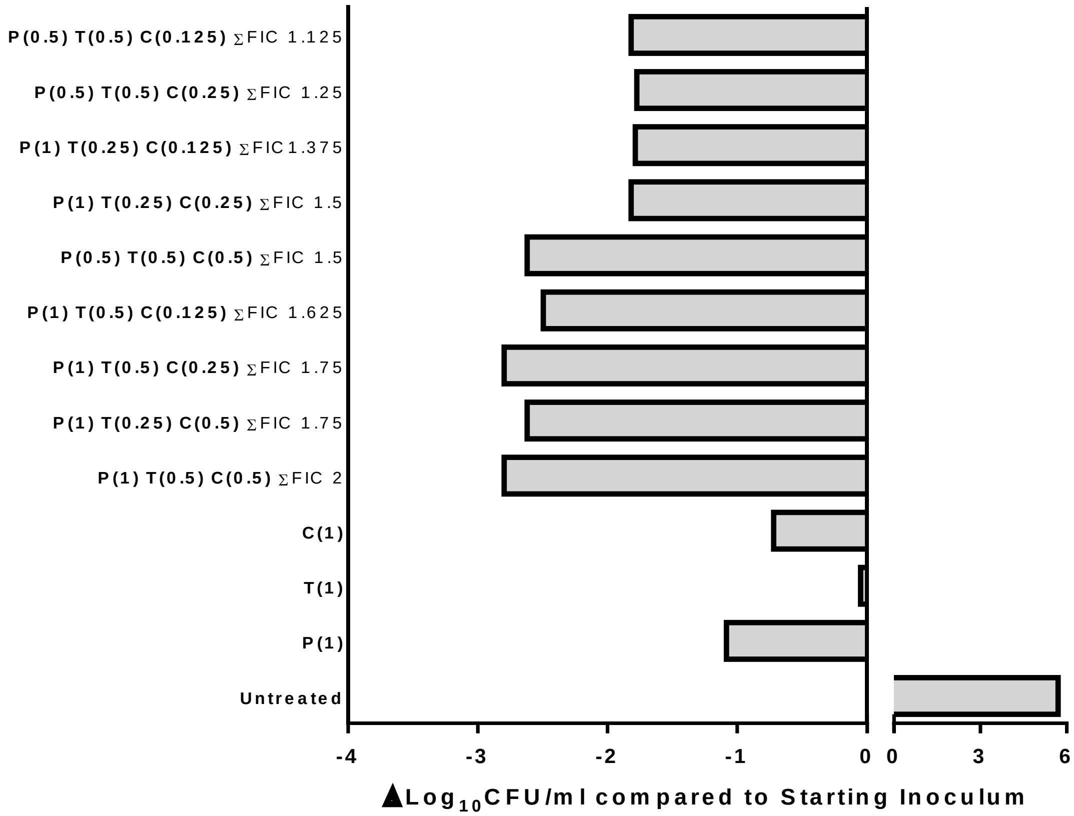

Table S1: Data from 51 Triplets; in silico FIC Analysis where the ΣFIC for each triplet across the various concentrations where Biomass arrest was observed in the platform.

**S. No.**	**Drug-A**	**Drug-B**	**Drug-C**	**∑FIC**
1	AMI	EMB	THI	∑FIC
	0.1	0.2	0.3	0.6
	0.1	0.3	0.3	0.7
	0.2	0.2	0.3	0.7
	0.2	0.3	0.2	0.7
	0.2	0.3	0.3	0.8
	0.3	0.2	0.3	0.8
	0.3	0.3	0.2	0.8
	0.3	0.3	0.3	0.9
2	AMI	MEM	MXF	∑FIC
	0.1	0.3	0.2	0.6
	0.1	0.3	0.3	0.7
	0.2	0.3	0.2	0.7
	0.2	0.3	0.3	0.8
	0.3	0.3	0.2	0.8
	0.3	0.3	0.3	0.9
3	BTZ	CAP	MEM	∑FIC
	0.2	0.1	0.3	0.6
	0.2	0.2	0.3	0.7
	0.2	0.3	0.3	0.8
	0.3	0.1	0.2	0.6
	0.3	0.1	0.3	0.7
	0.3	0.2	0.2	0.7
	0.3	0.2	0.3	0.8
	0.3	0.3	0.2	0.8
	0.3	0.3	0.3	0.9
4	BTZ	CFZ	LZD	∑FIC
	0.1	0.4	0.4	0.9
	0.2	0.4	0.4	1
	0.3	0.4	0.4	1.1
	0.4	0.4	0.4	1.2
5	BTZ	EMB	SQ	∑FIC
	0.1	0.3	0.3	0.7
	0.2	0.2	0.3	0.7
	0.2	0.3	0.3	0.8
	0.3	0.2	0.3	0.8
	0.3	0.3	0.2	0.8

**S. No.**	**Drug-A**	**Drug-B**	**Drug-C**	**∑FIC**
	0.3	0.3	0.3	0.9
6	BTZ	KAN	MEM	∑FIC
	0.2	0.1	0.3	0.6
	0.2	0.2	0.3	0.7
	0.2	0.3	0.3	0.8
	0.3	0.1	0.2	0.6
	0.3	0.1	0.3	0.7
	0.3	0.2	0.2	0.7
	0.3	0.2	0.3	0.8
	0.3	0.3	0.2	0.8
	0.3	0.3	0.3	0.9
7	BTZ	MEM	BDQ	∑FIC
	0.2	0.3	0.1	0.6
	0.2	0.3	0.2	0.7
	0.2	0.3	0.3	0.8
	0.3	0.2	0.1	0.6
	0.3	0.2	0.2	0.7
	0.3	0.2	0.3	0.8
	0.3	0.3	0.1	0.7
	0.3	0.3	0.2	0.8
	0.3	0.3	0.3	0.9
8	BTZ	MXF	SQ	∑FIC
	0.1	0.3	0.3	0.7
	0.2	0.3	0.3	0.8
	0.3	0.3	0.3	0.9
9	BTZ	MXF	THI	∑FIC
	0.1	0.3	0.3	0.7
	0.3	0.3	0.3	0.9
10	BTZ	PA824	BDQ	∑FIC
	0.1	0.3	0.4	0.8
	0.1	0.4	0.3	0.8
	0.1	0.4	0.4	0.9
	0.2	0.3	0.4	0.9
	0.2	0.4	0.3	0.9
	0.2	0.4	0.4	1
	0.3	0.3	0.4	1
	0.3	0.4	0.3	1

S. No.	Drug-A	Drug-B	Drug-C	∑FIC
	0.3	0.4	0.4	1.1
	0.4	0.3	0.4	1.1
	0.4	0.4	0.3	1.1
	0.4	0.4	0.4	1.2
11	BTZ	SQ	THI	∑FIC
	0.1	0.2	0.2	0.5
	0.2	0.2	0.1	0.5
	0.2	0.2	0.2	0.6
12	CLA	SQ	THI	∑FIC
	0.1	0.2	0.2	0.5
	0.2	0.2	0.2	0.6
13	CFZ	EMB)	PA824	∑FIC
	0.4	0.1	0.4	0.9
	0.4	0.2	0.4	1
	0.4	0.3	0.4	1.1
	0.4	0.4	0.4	1.2
14	CFZ	EMB	SQ	∑FIC
	0.1	0.3	0.3	0.7
	0.2	0.2	0.3	0.7
	0.2	0.3	0.3	0.8
	0.3	0.2	0.3	0.8
	0.3	0.3	0.3	0.9
15	CFZ	EMB	THI	∑FIC
	0.01	0.2	0.3	0.51
	0.01	0.3	0.3	0.61
	0.025	0.2	0.3	0.525
	0.025	0.3	0.3	0.625
	0.05	0.2	0.3	0.55
	0.05	0.3	0.3	0.65
	0.1	0.2	0.3	0.6
	0.1	0.3	0.3	0.7
	0.2	0.2	0.3	0.7
	0.2	0.3	0.2	0.7
	0.2	0.3	0.3	0.8
	0.3	0.2	0.3	0.8
	0.3	0.3	0.2	0.8
	0.3	0.3	0.3	0.9
16	CFZ	MXF	SQ	∑FIC
	0.1	0.2	0.4	0.7
	0.1	0.3	0.4	0.8

S. No.	Drug-A	Drug-B	Drug-C	∑FIC
	0.1	0.4	0.3	0.8
	0.1	0.4	0.4	0.9
	0.2	0.2	0.4	0.8
	0.2	0.3	0.4	0.9
	0.2	0.4	0.3	0.9
	0.2	0.4	0.4	1
	0.3	0.2	0.4	0.9
	0.3	0.3	0.4	1
	0.3	0.4	0.3	1
	0.3	0.4	0.4	1.1
	0.4	0.2	0.4	1
	0.4	0.3	0.4	1.1
	0.4	0.4	0.3	1.1
	0.4	0.4	0.4	1.2
17	CFZ	LZD	BDQ	∑FIC
	0.3	0.3	0.4	1
	0.3	0.4	0.3	1
	0.3	0.4	0.4	1.1
	0.4	0.1	0.4	0.9
	0.4	0.2	0.3	0.9
	0.4	0.2	0.4	1
	0.4	0.3	0.1	0.8
	0.4	0.3	0.2	0.9
	0.4	0.3	0.3	1
	0.4	0.3	0.4	1.1
	0.4	0.4	0.1	0.9
	0.4	0.4	0.2	1
	0.4	0.4	0.3	1.1
	0.4	0.4	0.4	1.2
18	CFZ	MXF	THI	∑ FIC
	0.1	0.2	0.4	0.7
	0.1	0.3	0.4	0.8
	0.1	0.4	0.3	0.8
	0.1	0.4	0.4	0.9
	0.2	0.2	0.4	0.8
	0.2	0.3	0.4	0.9
	0.2	0.4	0.3	0.9
	0.2	0.4	0.4	1
	0.3	0.2	0.4	0.9
	0.3	0.3	0.4	1

**S. No.**	**Drug-A**	**Drug-B**	**Drug-C**	**∑FIC**
	0.3	0.4	0.3	1
	0.3	0.4	0.4	1.1
	0.4	0.2	0.4	1
	0.4	0.3	0.4	1.1
	0.4	0.4	0.3	1.1
	0.4	0.4	0.4	1.2
19	CFZ	PA824	SQ	∑FIC
	0.4	0.3	0.1	0.8
	0.4	0.3	0.2	0.9
	0.4	0.3	0.3	1
	0.4	0.3	0.4	1.1
	0.4	0.4	0.1	0.9
	0.4	0.4	0.2	1
	0.4	0.4	0.3	1.1
	0.4	0.4	0.4	1.2
20	CFZ	PA824	THI	∑FIC
	0.1	0.4	0.4	0.9
	0.2	0.3	0.4	0.9
	0.2	0.4	0.4	1
	0.3	0.2	0.4	0.9
	0.3	0.3	0.4	1
	0.3	0.4	0.4	1.1
	0.4	0.2	0.4	1
	0.4	0.3	0.1	0.8
	0.4	0.3	0.2	0.9
	0.4	0.3	0.3	1
	0.4	0.3	0.4	1.1
	0.4	0.4	0.1	0.9
	0.4	0.4	0.2	1
	0.4	0.4	0.3	1.1
	0.4	0.4	0.4	1.2
21	CFZ	PA824	BDQ	∑FIC
	0.1	0.3	0.3	0.7
	0.2	0.2	0.3	0.7
	0.2	0.3	0.2	0.7
	0.2	0.3	0.3	0.8
	0.3	0.2	0.2	0.7
	0.3	0.2	0.3	0.8
	0.3	0.3	0.1	0.7
	0.3	0.3	0.2	0.8

**S. No.**	**Drug-A**	**Drug-B**	**Drug-C**	**∑FIC**
	0.3	0.3	0.3	0.9
22	CFZ	SQ)	THI	∑FIC
	0.1	0.1	0.2	0.4
	0.1	0.2	0.2	0.5
	0.2	0.2	0.2	0.6
23	DCS	EMB	SQ	∑FIC
	0.4	0.3	0.4	1.1
	0.4	0.4	0.3	1.1
	0.4	0.4	0.4	1.2
	0.1	0.3	0.4	0.8
	0.1	0.4	0.3	0.8
	0.1	0.4	0.4	0.9
	0.2	0.3	0.4	0.9
	0.2	0.4	0.3	0.9
	0.2	0.4	0.4	1
	0.3	0.3	0.4	1
	0.3	0.4	0.3	1
	0.3	0.4	0.4	1.1
24	DCS	EMB	THI	∑FIC
	0.1	0.3	0.3	0.7
	0.2	0.3	0.3	0.8
	0.3	0.3	0.3	0.9
25	DCS	MXF	SQ	∑FIC
	0.1	0.3	0.4	0.8
	0.1	0.4	0.3	0.8
	0.1	0.4	0.4	0.9
	0.4	0.2	0.4	1
	0.4	0.3	0.4	1.1
	0.4	0.4	0.3	1.1
	0.4	0.4	0.4	1.2
	0.1	0.3	0.4	0.8
	0.1	0.4	0.3	0.8
	0.1	0.4	0.4	0.9
	0.2	0.2	0.4	0.8
	0.2	0.3	0.4	0.9
	0.2	0.4	0.3	0.9
	0.2	0.4	0.4	1
	0.3	0.2	0.4	0.9
	0.3	0.3	0.4	1
	0.3	0.4	0.3	1

S. No.	Drug-A	Drug-B	Drug-C	∑FIC
	0.3	0.4	0.4	1.1
26	DCS	MXF	THI	∑FIC
	0.1	0.3	0.4	0.8
	0.1	0.4	0.3	0.8
	0.1	0.4	0.4	0.9
	0.4	0.2	0.4	1
	0.4	0.3	0.4	1.1
	0.4	0.4	0.3	1.1
	0.4	0.4	0.4	1.2
	0.1	0.3	0.4	0.8
	0.1	0.4	0.3	0.8
	0.1	0.4	0.4	0.9
	0.2	0.3	0.4	0.9
	0.2	0.4	0.3	0.9
	0.2	0.4	0.4	1
	0.3	0.3	0.4	1
	0.3	0.4	0.3	1
	0.3	0.4	0.4	1.1
27	DCS	PA824	BDQ	∑FIC
	0.1	0.4	0.4	0.9
	0.4	0.3	0.4	1.1
	0.4	0.4	0.3	1.1
	0.4	0.4	0.4	1.2
	0.1	0.3	0.4	0.8
	0.1	0.4	0.3	0.8
	0.1	0.4	0.4	0.9
	0.2	0.3	0.4	0.9
	0.2	0.4	0.3	0.9
	0.2	0.4	0.4	1
	0.3	0.3	0.4	1
	0.3	0.4	0.3	1
	0.3	0.4	0.4	1.1
28	DCS	SQ	THI	∑FIC
	0.1	0.1	0.2	0.4
	0.1	0.2	0.2	0.5
	0.2	0.2	0.2	0.6
29	EMB	LZD	SQ	∑FIC
	0.2	0.3	0.3	0.8
	0.3	0.1	0.2	0.6
	0.3	0.1	0.3	0.7

S. No.	Drug-A	Drug-B	Drug-C	∑FIC
	0.3	0.2	0.3	0.8
	0.3	0.3	0.3	0.9
30	EMB	LZD	THI	∑FIC
	0.2	0.1	0.3	0.6
	0.2	0.2	0.3	0.7
	0.2	0.3	0.3	0.8
	0.3	0.1	0.3	0.7
	0.3	0.2	0.2	0.7
	0.3	0.2	0.3	0.8
	0.3	0.3	0.2	0.8
	0.3	0.3	0.3	0.9
31	EMB	MXF	SQ	∑FIC
	0.1	0.3	0.4	0.8
	0.1	0.4	0.4	0.9
	0.3	0.3	0.4	1
	0.3	0.4	0.4	1.1
	0.4	0.1	0.4	0.9
	0.4	0.2	0.4	1
	0.4	0.3	0.4	1.1
	0.4	0.4	0.3	1.1
	0.4	0.4	0.4	1.2
32	EMB	MXF	THI	∑FIC
	0.1	0.3	0.4	0.8
	0.1	0.4	0.4	0.9
	0.3	0.1	0.4	0.8
	0.3	0.3	0.4	1
	0.3	0.4	0.4	1.1
	0.4	0.1	0.4	0.9
	0.4	0.2	0.4	1
	0.4	0.3	0.4	1.1
	0.4	0.4	0.4	1.2
33	EMB	PA824	SQ	∑ FIC
	0.2	0.1	0.3	0.6
	0.2	0.2	0.3	0.7
	0.2	0.3	0.3	0.8
	0.3	0.1	0.3	0.7
	0.3	0.2	0.3	0.8
	0.3	0.3	0.3	0.9
34	EMB	PA824	THI	∑FIC
	0.1	0.3	0.3	0.7

S. No.	Drug-A	Drug-B	Drug-C	∑FIC
	0.2	0.1	0.3	0.6
	0.2	0.2	0.3	0.7
	0.2	0.3	0.2	0.7
	0.2	0.3	0.3	0.8
	0.3	0.1	0.2	0.6
	0.3	0.1	0.3	0.7
	0.3	0.2	0.2	0.7
	0.3	0.2	0.3	0.8
	0.3	0.3	0.2	0.8
	0.3	0.3	0.3	0.9
35	EMB	PA824	BDQ	∑FIC
	0.1	0.3	0.4	0.8
	0.1	0.4	0.3	0.8
	0.1	0.4	0.4	0.9
	0.2	0.3	0.4	0.9
	0.2	0.4	0.3	0.9
	0.2	0.4	0.4	1
	0.3	0.2	0.4	0.9
	0.3	0.3	0.4	1
	0.3	0.4	0.3	1
	0.3	0.4	0.4	1.1
	0.4	0.2	0.4	1
	0.4	0.3	0.3	1
	0.4	0.3	0.4	1.1
	0.4	0.4	0.2	1
	0.4	0.4	0.3	1.1
	0.4	0.4	0.4	1.2
36	EMB	SQ	THI	∑FIC
	0.1	0.1	0.2	0.4
	0.1	0.2	0.1	0.4
	0.1	0.2	0.2	0.5
	0.2	0.1	0.1	0.4
	0.2	0.1	0.2	0.5
	0.2	0.2	0.1	0.5
	0.2	0.2	0.2	0.6
37	EMB	SQ	BDQ	∑FIC
	0.3	0.3	0.1	0.7
	0.3	0.3	0.2	0.8
	0.3	0.3	0.3	0.9
38	EMB	BDQ	THI	∑FIC

S. No.	Drug-A	Drug-B	Drug-C	∑FIC
	0.2	0.1	0.3	0.6
	0.2	0.2	0.3	0.7
	0.2	0.3	0.3	0.8
	0.3	0.1	0.3	0.7
	0.3	0.2	0.3	0.8
	0.3	0.3	0.3	0.9
39	KAN	SQ	THI	∑FIC
	0.1	0.1	0.2	0.4
	0.1	0.2	0.2	0.5
	0.2	0.2	0.2	0.6
40	LZD	MXF	THI	∑FIC
	0.1	0.2	0.4	0.7
	0.1	0.3	0.4	0.8
	0.1	0.4	0.3	0.8
	0.1	0.4	0.4	0.9
	0.2	0.2	0.4	0.8
	0.2	0.3	0.4	0.9
	0.2	0.4	0.3	0.9
	0.2	0.4	0.4	1
	0.3	0.2	0.4	0.9
	0.3	0.3	0.4	1
	0.3	0.4	0.3	1
	0.3	0.4	0.4	1.1
	0.4	0.2	0.4	1
	0.4	0.3	0.4	1.1
	0.4	0.4	0.3	1.1
	0.4	0.4	0.4	1.2
41	LZD	MXF	SQ	∑FIC
	0.1	0.2	0.4	0.7
	0.1	0.3	0.3	0.7
	0.1	0.3	0.4	0.8
	0.1	0.4	0.3	0.8
	0.1	0.4	0.4	0.9
	0.2	0.2	0.4	0.8
	0.2	0.3	0.4	0.9
	0.2	0.4	0.3	0.9
	0.2	0.4	0.4	1
	0.3	0.2	0.4	0.9
	0.3	0.3	0.4	1
	0.3	0.4	0.3	1

S. No.	Drug-A	Drug-B	Drug-C	∑FIC
	0.3	0.4	0.4	1.1
	0.4	0.2	0.4	1
	0.4	0.3	0.4	1.1
	0.4	0.4	0.3	1.1
	0.4	0.4	0.4	1.2
42	LZD	SQ	THI	∑FIC
	0.1	0.1	0.2	0.45
	0.1	0.2	0.2	0.5
	0.2	0.2	0.2	0.6
43	MEM	SQ	THI	∑FIC
	0.1	0.2	0.1	0.4
	0.1	0.2	0.2	0.5
	0.2	0.1	0.2	0.5
	0.2	0.2	0.1	0.5
	0.2	0.2	0.2	0.6
44	MXF	PA824	SQ	∑FIC
	0.2	0.1	0.4	0.7
	0.2	0.2	0.4	0.8
	0.2	0.3	0.4	0.9
	0.3	0.01	0.3	0.61
	0.3	0.1	0.3	0.7
	0.3	0.1	0.4	0.8
	0.3	0.2	0.4	0.9
	0.3	0.3	0.4	1
	0.3	0.4	0.4	1.1
	0.4	0.1	0.3	0.8
	0.4	0.1	0.4	0.9
	0.4	0.2	0.3	0.9
	0.4	0.2	0.4	1
	0.4	0.3	0.4	1.1
	0.4	0.4	0.4	1.2
45	MXF	PA824	THI	∑FIC
	0.3	0.1	0.3	0.7
	0.3	0.2	0.3	0.8
	0.3	0.3	0.3	0.9
46	MXF	PA824	BDQ	∑FIC
	0.2	0.4	0.4	1
	0.3	0.4	0.4	1.1
	0.4	0.4	0.4	1.2
47	MXF	SQ	THI	∑FIC

S. No.	Drug-A	Drug-B	Drug-C	∑FIC
	0.1	0.1	0.2	0.4
	0.1	0.2	0.1	0.4
	0.1	0.2	0.2	0.5
	0.2	0.1	0.2	0.5
	0.2	0.2	0.1	0.5
	0.2	0.2	0.2	0.6
48	PA824	SQ	BDQ	∑FIC
	0.2	0.4	0.4	1
	0.3	0.1	0.4	0.8
	0.3	0.2	0.4	0.9
	0.3	0.3	0.4	1
	0.3	0.4	0.3	1
	0.3	0.4	0.4	1.1
	0.4	0.1	0.3	0.8
	0.4	0.1	0.4	0.9
	0.4	0.2	0.3	0.9
	0.4	0.2	0.4	1
	0.4	0.3	0.3	1
	0.4	0.3	0.4	1.1
	0.4	0.4	0.3	1.1
	0.4	0.4	0.4	1.2
49	PA824	SQ	THI	∑FIC
	0.1	0.2	0.1	0.4
	0.1	0.2	0.2	0.5
	0.2	0.1	0.2	0.5
	0.2	0.2	0.1	0.5
	0.2	0.2	0.2	0.6
50	SQ	STR	THI	∑FIC
	0.1	0.1	0.2	0.4
	0.2	0.1	0.2	0.5
	0.2	0.2	0.2	0.6
51	SQ	BDQ	THI	∑FIC
	0.1	0.2	0.2	0.5
	0.2	0.2	0.1	0.5
	0.2	0.1	0.2	0.5
	0.2	0.2	0.2	0.6

Table S2: In vitro ∑FIC and bacterial reduction (ΔLog_{10}CFU/ml) for each triplet. The lanes marked “BS” indicate the best synergistic concentrations for a particular triplet, with each drug concentration not less than 0.1 FIC i.e., 1/10th MIC. The lanes marked “BC” indicate the maximum bacterial kill achieved, with each drug concentration not greater than 0.5 FIC i.e., ½ MIC.

**S. No.**	**A**	**B**	**C**	**ΣFIC**	**ΔLog$_{10}$CFU/ml**
**Ref**	**INH**	**RIF**	**EMB**	**ΣFIC**	**ΔLog$_{10}$CFU/ml**
BS	0.125	0.5	0.125	0.75	-0.3
	0.125	0.5	0.25	0.88	-0.3
	0.25	0.5	0.125	0.88	-0.3
	0.25	0.5	0.25	1	-0.3
	0.125	0.5	0.5	1.13	-0.3
	0.5	0.5	0.125	1.13	-0.3
	0.25	0.5	0.5	1.25	-0.3
	0.5	0.5	0.25	1.25	-0.3
	0.5	0.5	0.5	1.5	-0.3
**Ref**	**INH**	**RIF**	**EMB**	**ΣFIC**	**ΔLog$_{10}$CFU/ml**
BS	0.125	0.5	0.125	0.75	-0.3
	0.25	0.5	0.125	0.875	-0.3
	0.25	0.5	0.25	1	-0.3
	0.125	0.5	0.5	1.125	-0.3
	0.5	0.5	0.125	1.125	-0.3
	0.25	0.5	0.5	1.25	-0.3
	0.5	0.5	0.25	1.25	-0.3
	0.5	0.5	0.5	1.5	-0.3
**1**	**AMI**	**EMB**	**THI**	**ΣFIC**	**ΔLog$_{10}$CFU/ml**
BS	0.5	0.5	0.125	1.125	-0.3

**S. No.**	**A**	**B**	**C**	**ΣFIC**	**ΔLog$_{10}$CFU/ml**
	0.5	0.5	0.25	1.25	-0.3
BC	0.5	0.5	0.5	1.5	-0.3
**2**	**AMI**	**MEM**	**MXF**	**ΣFIC**	**ΔLog$_{10}$CFU/ml**
BS	0.125	0.5	0.125	0.75	-0.3
	0.125	0.5	0.25	0.875	0.76
	0.5	0.25	0.125	0.875	0.4
	0.25	0.5	0.125	0.875	0.32
	0.25	0.5	0.25	1	0.61
	0.5	0.25	0.25	1	0.59
	0.5	0.5	0.125	1.125	1.19
BC	0.5	0.5	0.25	1.25	1.44
**3**	**BTZ**	**CAP**	**MEM**	**ΣFIC**	**ΔLog$_{10}$CFU/ml**
BS	0.125	0.25	0.5	0.875	-0.3
	0.25	0.25	0.5	1	-0.3
	0.5	0.125	0.5	1.125	0.94
	0.125	0.5	0.5	1.125	-0.3
	0.5	0.25	0.5	1.25	1.02
	0.5	0.5	0.25	1.25	0.79
	0.25	0.5	0.5	1.25	0
BC	0.5	0.5	0.5	1.5	1.52
**4**	**BTZ**	**CFZ**	**LZD**	**ΣFIC**	**ΔLog$_{10}$CFU/ml**

S. No.	A	B	C	ΣFIC	$\Delta Log_{10}CFU/ml$
BS	0.125	0.5	0.125	0.75	0.27
	0.125	0.5	0.25	0.875	0.27
	0.25	0.5	0.125	0.875	0.27
	0.5	0.5	0.125	1.125	1.3
	0.5	0.5	0.25	1.25	1.74
BC	0.5	0.5	0.5	1.5	1.87
**5**	**BTZ**	**EMB**	**SQ**	**ΣFIC**	**$\Delta Log_{10}CFU/ml$**
BS	0.5	0.125	0.125	0.75	1.4
	0.5	0.125	0.25	0.875	2.22
	0.5	0.25	0.125	0.875	1.62
	0.125	0.5	0.25	0.875	1.4
	0.25	0.5	0.125	0.875	1.22
	0.5	0.25	0.25	1	2.36
	0.25	0.5	0.25	1	1.8
	0.5	0.125	0.5	1.125	3.7
	0.5	0.5	0.125	1.125	2.66
	0.125	0.5	0.5	1.125	2
	0.5	0.25	0.5	1.25	3.7
	0.5	0.5	0.25	1.25	2.7
	0.25	0.5	0.5	1.25	2.22
BC	0.5	0.5	0.5	1.5	3.7
**6**	**BTZ**	**KAN**	**MEM**	**ΣFIC**	**$\Delta Log_{10}CFU/ml$**
BS	0.125	0.125	0.5	0.75	-0.3
	0.125	0.25	0.5	0.875	-0.3
	0.25	0.125	0.5	0.875	-0.3
	0.5	0.25	0.25	1	0.3

S. No.	A	B	C	ΣFIC	$\Delta Log_{10}CFU/ml$
	0.25	0.25	0.5	1	0
	0.5	0.125	0.5	1.125	1.02
	0.125	0.5	0.5	1.125	0.49
	0.5	0.25	0.5	1.25	1.28
	0.25	0.5	0.5	1.25	0.79
	0.5	0.5	0.25	1.25	0.47
BC	0.5	0.5	0.5	1.5	1.42
**7**	**BTZ**	**MEM**	**TMC**	**ΣFIC**	**$\Delta Log_{10}CFU/ml$**
BS	0.125	0.5	0.125	0.75	0
	0.5	0.25	0.125	0.875	1.42
	0.25	0.5	0.125	0.875	0.87
	0.5	0.125	0.25	0.875	0.75
	0.125	0.5	0.25	0.875	0.74
	0.5	0.25	0.25	1	1.74
	0.25	0.5	0.25	1	1.32
	0.25	0.25	0.5	1	1.05
	0.5	0.125	0.5	1.125	1.38
	0.125	0.5	0.5	1.125	1.11
	0.25	0.5	0.5	1.25	1.3
	0.5	0.25	0.5	1.25	1.24
BC	0.5	0.5	0.5	1.5	1.38
**8**	**BTZ**	**MXF**	**SQ**	**ΣFIC**	**$\Delta Log_{10}CFU/ml$**
BS	0.125	0.5	0.125	0.75	0.27
	0.5	0.125	0.25	0.875	2.92
	0.5	0.25	0.125	0.875	2.25
	0.125	0.5	0.25	0.875	0.27

S. No.	A	B	C	ΣFIC	ΔLog_{10}CFU/ml
	0.25	0.5	0.125	0.875	0.27
	0.5	0.25	0.25	1	2.92
	0.25	0.5	0.25	1	1.62
	0.5	0.125	0.5	1.125	3.07
	0.5	0.5	0.125	1.125	2.92
	0.125	0.5	0.5	1.125	2.32
	0.5	0.25	0.5	1.25	3.1
	0.5	0.5	0.25	1.25	3.2
	0.25	0.5	0.5	1.25	2.92
BC	0.5	0.5	0.5	1.5	3.2
**9**	**BTZ**	**MXF**	**THI**	**ΣFIC**	**ΔLog_{10}CFU/ml**
BS	0.125	0.5	0.25	0.875	0.27
	0.25	0.5	0.25	1	0.27
	0.5	0.5	0.125	1.125	0.27
BC	0.5	0.5	0.25	1.25	0.7
**10**	**BTZ**	**PA824**	**TMC**	**ΣFIC**	**ΔLog_{10}CFU/ml**
BS	0.125	0.125	0.5	0.75	2.44
	0.5	0.125	0.125	0.75	1.21
	0.125	0.25	0.5	0.875	2.85
	0.25	0.125	0.5	0.875	2.8
	0.5	0.125	0.25	0.875	2.62
	0.125	0.5	0.25	0.875	2
	0.5	0.25	0.125	0.875	1.34
	0.25	0.5	0.125	0.875	0.3
	0.25	0.25	0.5	1	2.62
	0.5	0.25	0.25	1	2.55

S. No.	A	B	C	ΣFIC	ΔLog_{10}CFU/ml
	0.25	0.5	0.25	1	2.1
	0.5	0.125	0.5	1.125	2.7
	0.125	0.5	0.5	1.125	2.38
	0.5	0.5	0.125	1.125	1.62
	0.5	0.5	0.25	1.25	2.92
	0.5	0.25	0.5	1.25	2.62
	0.25	0.5	0.5	1.25	2.12
BC	0.5	0.5	0.5	1.5	3.44
**11**	**BTZ**	**SQ**	**THI**	**ΣFIC**	**ΔLog_{10}CFU/ml**
BS	0.125	0.5	0.125	0.75	2.06
	0.125	0.5	0.25	0.875	2.14
	0.25	0.5	0.125	0.875	2.08
	0.5	0.25	0.125	0.875	1
	0.25	0.5	0.25	1	2.21
	0.5	0.25	0.25	1	1.92
	0.5	0.5	0.125	1.125	2.49
BC	0.5	0.5	0.25	1.25	2.7
**12**	**CLA**	**SQ**	**THI**	**ΣFIC**	**ΔLog_{10}CFU/ml**
BS	0.125	0.5	0.125	0.75	1.3
	0.125	0.125	0.5	0.75	1.14
	0.25	0.25	0.25	0.75	0.68
	0.125	0.5	0.25	0.875	2.22
	0.25	0.5	0.125	0.875	1.85
	0.125	0.25	0.5	0.875	1.52
	0.25	0.125	0.5	0.875	0.67
	0.25	0.5	0.25	1	2.7

S. No.	A	B	C	ΣFIC	ΔLog$_{10}$CFU/ml
	0.25	0.25	0.5	1	1.42
	0.125	0.5	0.5	1.125	2.1
BC	0.25	0.5	0.5	1.25	2.7
**13**	**CFZ**	**EMB**	**PA824**	**ΣFIC**	**ΔLog$_{10}$CFU/ml**
BS	0.125	0.5	0.25	0.875	0.3
	0.25	0.5	0.25	1	0.3
	0.125	0.5	0.5	1.125	0.3
	0.25	0.5	0.5	1.25	0.3
	0.5	0.5	0.25	1.25	0.3
BC	0.5	0.5	0.5	1.5	0.3
**14**	**CFZ**	**EMB**	**SQ**	**ΣFIC**	**ΔLog$_{10}$CFU/ml**
BS	0.5	0.125	0.125	0.75	0.7
	0.5	0.25	0.125	0.875	1
	0.25	0.125	0.5	0.875	0.27
	0.5	0.125	0.25	0.875	0.27
	0.25	0.25	0.5	1	1.52
	0.5	0.25	0.25	1	0.27
	0.5	0.125	0.5	1.125	1.3
	0.5	0.5	0.125	1.125	1
	0.25	0.5	0.5	1.25	1.76
	0.5	0.5	0.25	1.25	1.62
	0.5	0.25	0.5	1.25	1.59
	1	0.125	0.25	1.375	1
BC	0.5	0.5	0.5	1.5	1.63
**15**	**CFZ**	**EMB**	**THI**	**ΣFIC**	**ΔLog$_{10}$CFU/ml**
BS	0.5	0.125	0.5	1.125	0.25

S. No.	A	B	C	ΣFIC	ΔLog$_{10}$CFU/ml
	0.5	0.5	0.125	1.125	0.25
	0.5	0.25	0.5	1.25	0.25
	0.5	0.5	0.25	1.25	0.25
BC	0.5	0.5	0.5	1.5	0.25
**16**	**CFZ**	**LZD**	**TMC**	**ΣFIC**	**ΔLog$_{10}$CFU/ml**
BS	0.25	0.125	0.5	0.875	0.27
	0.25	0.25	0.5	1	0.27
	0.5	0.125	0.5	1.125	0.27
	0.125	0.5	0.5	1.125	0.27
	0.5	0.25	0.5	1.25	0.27
	0.25	0.5	0.5	1.25	0.27
BC	0.5	0.5	0.5	1.5	0.27
**17**	**CFZ**	**MXF**	**SQ**	**ΣFIC**	**ΔLog$_{10}$CFU/ml**
BS	0.5	0.25	0.5	1.25	1
	0.5	0.5	0.5	1.5	1
BC	0.5	0.125	0.5	1.125	0.7
**18**	**CFZ**	**MXF**	**THI**	**ΣFIC**	**ΔLog$_{10}$CFU/ml**
BS	0.125	0.125	0.5	0.75	-0.3
	0.25	0.125	0.5	0.875	-0.3
	0.125	0.25	0.5	0.875	-0.3
	0.125	0.5	0.25	0.875	-0.3
	0.25	0.25	0.5	1	-0.3
	0.25	0.5	0.25	1	-0.3
	0.125	0.5	0.5	1.125	0.38
	0.5	0.125	0.5	1.125	-0.3
	0.5	0.5	0.125	1.125	-0.3

S. No.	A	B	C	ΣFIC	ΔLog$_{10}$CFU/ml
	0.25	0.5	0.5	1.25	0.51
	0.5	0.25	0.5	1.25	-0.3
	0.5	0.5	0.25	1.25	-0.3
BC	0.5	0.5	0.5	1.5	0.65
**19**	**CFZ**	**PA824**	**SQ**	**ΣFIC**	**ΔLog$_{10}$CFU/ml**
BS	0.125	0.25	0.5	0.875	2.4
	0.125	0.5	0.25	0.875	2.06
	0.25	0.25	0.5	1	2.49
	0.25	0.5	0.25	1	2.17
	0.06	0.5	0.5	1.06	3.7
	0.5	0.06	0.5	1.06	1.3
	0.125	0.5	0.5	1.125	3.4
	0.5	0.125	0.5	1.125	1.18
	0.25	0.5	0.5	1.25	3.7
	0.5	0.5	0.25	1.25	2.08
	0.5	0.25	0.5	1.25	2.06
BC	0.5	0.5	0.5	1.5	3.7
**20**	**CFZ**	**PA824**	**THI**	**ΣFIC**	**ΔLog$_{10}$CFU/ml**
BS	0.5	0.125	0.125	0.75	0.27
	0.125	0.5	0.25	0.875	3.1
	0.5	0.125	0.25	0.875	0.27
	0.5	0.25	0.125	0.875	0.27
	0.25	0.5	0.25	1	3.1
	0.5	0.25	0.25	1	0.27
	0.5	0.5	0.125	1.125	0.27
BC	0.5	0.5	0.25	1.25	3.4

S. No.	A	B	C	ΣFIC	ΔLog$_{10}$CFU/ml
**21**	**CFZ**	**PA824**	**TMC**	**ΣFIC**	**ΔLog$_{10}$CFU/ml**
BS	0.125	0.5	0.5	1.125	2.12
	0.5	0.125	0.5	1.125	1.25
	0.25	0.5	0.5	1.25	2.08
	0.5	0.25	0.5	1.25	1.25
BC	0.5	0.5	0.5	1.5	2.06
**22**	**CFZ**	**SQ**	**THI**	**ΣFIC**	**ΔLog$_{10}$CFU/ml**
BS	0.125	0.5	0.125	0.75	1.22
	0.25	0.25	0.25	0.75	0.3
	0.5	0.125	0.125	0.75	0.3
	0.25	0.5	0.125	0.875	2
	0.125	0.5	0.25	0.875	1.19
	0.5	0.125	0.25	0.875	1.1
	0.5	0.25	0.125	0.875	1.09
	0.25	0.5	0.25	1	2.1
	0.5	0.25	0.25	1	1.19
	0.5	0.5	0.125	1.125	2.28
BC	0.5	0.5	0.25	1.25	2.7
**23**	**DCS**	**EMB**	**SQ**	**ΣFIC**	**ΔLog$_{10}$CFU/ml**
BS	0.125	0.5	0.5	1.125	3.22
	0.25	0.5	0.5	1.25	3.4
BC	0.5	0.5	0.5	1.5	3.7
**24**	**DCS**	**EMB**	**THI**	**ΣFIC**	**ΔLog$_{10}$CFU/ml**
BS	0.5	0.5	0.125	1.125	1.12
BC	0.5	0.5	0.25	1.25	1.28
**25**	**DCS**	**MXF**	**SQ**	**ΣFIC**	**ΔLog$_{10}$CFU/ml**
BS/BC	0.5	0.5	0.5	1.5	0.3

S. No.	A	B	C	ΣFIC	ΔLog_{10}CFU/ml
**26**	**DCS**	**MXF**	**THI**	**ΣFIC**	**ΔLog_{10}CFU/ml**
BS	0.5	0.5	0.125	1.125	0.3
BC	0.5	0.5	0.25	1.25	0.3
**27**	**DCS**	**PA824**	**TMC**	**ΣFIC**	**ΔLog_{10}CFU/ml**
BS	0.125	0.5	0.25	0.875	2.52
	0.25	0.5	0.25	1	2.24
	0.125	0.5	0.5	1.125	2.1
	0.5	0.5	0.25	1.25	2.25
	0.25	0.5	0.5	1.25	2.19
	0.5	0.25	0.5	1.25	1.98
	0.5	0.25	0.5	1.25	1.3
BC	0.5	0.5	0.5	1.5	2.92
**28**	**DCS**	**SQ**	**THI**	**ΣFIC**	**ΔLog_{10}CFU/ml**
BS	0.125	0.5	0.5	1.125	3.1
	0.25	0.5	0.5	1.25	3.1
	0.5	0.5	0.5	1.5	3.1
	0.125	0.5	0.25	0.875	1.15
	0.25	0.5	0.25	1	0.83
	0.125	0.5	0.5	1.125	2.7
	0.25	0.5	0.5	1.25	2.7
	0.5	0.5	0.25	1.25	0.81
BC	0.5	0.5	0.5	1.5	2.7
**29**	**EMB**	**LZD**	**SQ**	**ΣFIC**	**ΔLog_{10}CFU/ml**
BS	0.5	0.125	0.5	1.125	2.02
	0.125	0.5	0.5	1.125	1.89
	0.5	0.25	0.5	1.25	2.02

S. No.	A	B	C	ΣFIC	ΔLog_{10}CFU/ml
	0.25	0.5	0.5	1.25	2.08
BC	0.5	0.5	0.5	1.5	2.19
**30**	**EMB**	**LZD**	**THI**	**ΣFIC**	**ΔLog_{10}CFU/ml**
BS	0.5	0.5	0.25	1.25	0.27
BC	0.5	0.5	0.125	1.125	0.27
**31**	**EMB**	**SQ**	**MXF**	**ΣFIC**	**ΔLog_{10}CFU/ml**
BS	0.5	0.5	0.125	1.125	3.7
	0.5	0.5	0.25	1.125	3.7
BC	0.5	0.5	0.5	1.25	3.7
**32**	**EMB**	**MXF**	**THI**	**ΣFIC**	**ΔLog_{10}CFU/ml**
BS	0.5	0.5	0.125	1.125	0.3
BC	0.5	0.5	0.25	1.25	0.3
**33**	**EMB**	**PA824**	**SQ**	**ΣFIC**	**ΔLog_{10}CFU/ml**
BS	0.125	0.25	0.5	0.875	3.7
	0.25	0.5	0.125	0.875	3.4
	0.5	0.25	0.125	0.875	3
	0.125	0.5	0.25	0.875	2.92
	0.5	0.125	0.25	0.875	2.62
	0.25	0.5	0.25	1	3.7
	0.5	0.25	0.25	1	3.4
	0.5	0.125	0.5	1.125	3.7
	0.125	0.5	0.5	1.125	3.7
	0.5	0.5	0.125	1.125	2.92
	0.5	0.25	0.5	1.25	3.7
	0.25	0.5	0.5	1.25	3.7
	0.5	0.5	0.25	1.25	3.22

S. No.	A	B	C	ΣFIC	ΔLog_{10}CFU/ml
BC	0.5	0.5	0.5	1.5	3.7
**34**	**EMB**	**PA824**	**THI**	**ΣFIC**	**ΔLog_{10}CFU/ml**
BS	0.125	0.25	0.25	0.625	3.22
	0.25	0.25	0.25	0.75	3.7
	0.125	0.5	0.125	0.75	3.4
	0.125	0.5	0.25	0.875	3.7
	0.25	0.5	0.125	0.875	3.1
	0.25	0.5	0.25	1	3.7
	0.5	0.25	0.25	1	2.85
	0.5	0.5	0.125	1.125	3.22
BC	0.5	0.5	0.25	1.25	3.7
**35**	**EMB**	**PA824**	**TMC**	**ΣFIC**	**ΔLog_{10}CFU/ml**
BS	0.5	0.125	0.5	1.125	2.18
	0.125	0.5	0.5	1.125	2.02
	0.25	0.5	0.5	1.25	2.12
	0.5	0.25	0.5	1.25	2.12
BC	0.5	0.5	0.5	1.5	2.16
**36**	**EMB**	**SQ**	**THI**	**ΣFIC**	**ΔLog_{10}CFU/ml**
BS	0.125	0.25	0.5	0.875	0.45
	0.25	0.25	0.5	1	0.41
	0.125	0.5	0.5	1.125	1.3
	0.5	0.125	0.5	1.125	0.17
	0.25	0.5	0.5	1.25	1.66
	0.5	0.25	0.5	1.25	1.3
BC	0.5	0.5	0.5	1.5	2.7
**37**	**EMB**	**SQ**	**TMC**	**ΣFIC**	**ΔLog_{10}CFU/ml**

S. No.	A	B	C	ΣFIC	ΔLog_{10}CFU/ml
BS	0.125	0.5	0.25	0.875	1.75
	0.125	0.25	0.5	0.875	0.27
	0.25	0.5	0.25	1	2.03
	0.25	0.25	0.5	1	0.27
	0.5	0.5	0.06	1.06	2.32
	0.5	0.5	0.06	1.06	2.12
	0.5	0.5	0.125	1.125	3.7
	0.125	0.5	0.5	1.125	2.19
	0.5	0.125	0.5	1.125	1.74
	0.5	0.5	0.25	1.25	2.59
	0.25	0.5	0.5	1.25	2.3
	0.5	0.25	0.5	1.25	2.17
	0.5	0.5	0.25	1.25	2.1
BC	0.5	0.5	0.5	1.5	2.62
**38**	**EMB**	**THI**	**TMC**	**ΣFIC**	**ΔLog_{10}CFU/ml**
BS	0.125	0.125	0.5	0.75	0.27
	0.125	0.25	0.5	0.875	1.55
	0.25	0.125	0.5	0.875	1.27
	0.25	0.25	0.5	1	2.4
	0.5	0.125	0.5	1.125	2.27
BC	0.5	0.5	0.25	1.25	2.24
**39**	**KAN**	**SQ**	**THI**	**ΣFIC**	**ΔLog_{10}CFU/ml**
BS	0.125	0.5	0.25	0.875	0.59
	0.25	0.5	0.25	1	1.36
	0.125	0.5	0.5	1.125	2.1
	0.5	0.5	0.125	1.125	0

S. No.	A	B	C	ΣFIC	$\Delta Log_{10}CFU/ml$
	0.25	0.5	0.5	1.25	2.7
	0.5	0.5	0.25	1.25	1.62
BC	0.5	0.5	0.5	1.5	2.7
**40**	**LZD**	**MXF**	**SQ**	**ΣFIC**	**$\Delta Log_{10}CFU/ml$**
BS	0.5	0.5	0.125	1.125	0.25
	0.125	0.5	0.5	1.125	0.25
	0.5	0.5	0.25	1.25	0.25
	0.25	0.5	0.5	1.25	0.25
BC	0.5	0.5	0.5	1.5	0.25
**41**	**LZD**	**MXF**	**THI**	**ΣFIC**	**$\Delta Log_{10}CFU/ml$**
BS	0.25	0.125	0.5	0.875	0
	0.125	0.25	0.5	0.875	0
	0.25	0.5	0.125	0.875	-0.3
	0.125	0.5	0.25	0.875	-0.3
	0.25	0.25	0.5	1	0.32
	0.5	0.25	0.25	1	0
	0.25	0.5	0.25	1	-0.3
	0.125	0.5	0.5	1.125	0.75
	0.5	0.125	0.5	1.125	0
	0.5	0.5	0.125	1.125	-0.3
	0.5	0.25	0.5	1.25	0.49
	0.5	0.5	0.25	1.25	0.42
	0.25	0.5	0.5	1.25	0
BC	0.5	0.5	0.5	1.5	0.89
**42**	**LZD**	**SQ**	**THI**	**ΣFIC**	**$\Delta Log_{10}CFU/ml$**
BS/BC	0.5	0.5	0.25	1.25	1.85

S. No.	A	B	C	ΣFIC	$\Delta Log_{10}CFU/ml$
	0.25	0.5	0.25	1	0.3
**43**	**MEM**	**SQ**	**THI**	**ΣFIC**	**$\Delta Log_{10}CFU/ml$**
BS	0.125	0.5	0.125	0.75	2.7
	0.25	0.25	0.25	0.75	2
	0.125	0.125	0.5	0.75	0.87
	0.5	0.125	0.125	0.75	0.81
	0.5	0.25	0.125	0.875	2.7
	0.25	0.5	0.125	0.875	2.7
	0.125	0.5	0.25	0.875	2.4
	0.5	0.125	0.25	0.875	2
	0.125	0.25	0.5	0.875	2
	0.25	0.125	0.5	0.875	1.74
	0.5	0.25	0.25	1	2.7
	0.25	0.25	0.5	1	2.7
	0.25	0.5	0.25	1	2.7
	0.5	0.5	0.125	1.125	2.7
	0.125	0.5	0.5	1.125	2.7
	0.5	0.125	0.5	1.125	2.4
	0.5	0.25	0.5	1.25	2.7
	0.5	0.5	0.25	1.25	2.7
	0.25	0.5	0.5	1.25	2.7
BC	0.5	0.5	0.5	1.5	2.7
**44**	**MXF**	**PA824**	**SQ**	**ΣFIC**	**$\Delta Log_{10}CFU/ml$**
BS	0.125	0.125	0.5	0.75	1.97
	0.25	0.125	0.5	0.875	2.92
	0.125	0.25	0.5	0.875	2.92

S. No.	A	B	C	ΣFIC	$\Delta Log_{10}CFU/ml$
	0.125	0.5	0.25	0.875	1.98
	0.25	0.25	0.5	1	3.4
	0.5	0.25	0.25	1	2.66
	0.25	0.5	0.25	1	2.49
	0.5	0.125	0.5	1.125	3.7
	0.125	0.5	0.5	1.125	3.4
	0.5	0.5	0.125	1.125	2.49
	0.5	0.25	0.5	1.25	3.7
	0.25	0.5	0.5	1.25	3.7
	0.5	0.5	0.25	1.25	3.22
BC	0.5	0.5	0.5	1.5	3.4
**45**	**MXF**	**PA824**	**THI**	**ΣFIC**	**$\Delta Log_{10}CFU/ml$**
BS	0.125	0.25	0.25	0.625	3.7
	0.25	0.25	0.25	0.75	3.7
	0.125	0.5	0.25	0.875	3.7
	0.5	0.25	0.25	1	3.7
	0.25	0.5	0.25	1	3.7
BC	0.5	0.5	0.25	1.25	3.7
**46**	**MXF**	**PA824**	**TMC**	**ΣFIC**	**$\Delta Log_{10}CFU/ml$**
BS	0.25	0.25	0.5	1	1.67
	0.125	0.5	0.5	1.125	3
	0.5	0.125	0.5	1.125	1.55
	0.5	0.5	0.25	1.25	2.49
	0.25	0.5	0.5	1.25	2.36
BC	0.5	0.5	0.5	1.5	3.1
**47**	**MXF**	**SQ**	**THI**	**ΣFIC**	**$\Delta Log_{10}CFU/ml$**

S. No.	A	B	C	ΣFIC	$\Delta Log_{10}CFU/ml$
BS	0.5	0.5	0.125	1.125	3.22
BC	0.5	0.5	0.25	1.25	3.4
**48**	**PA824**	**SQ**	**THI**	**ΣFIC**	**$\Delta Log_{10}CFU/ml$**
BS	0.125	0.5	0.125	0.75	3.4
	0.125	0.5	0.25	0.875	3.7
	0.5	0.125	0.25	0.875	3.4
	0.25	0.5	0.125	0.875	3.4
	0.5	0.25	0.25	1	3.7
	0.25	0.5	0.25	1	3.7
	0.5	0.5	0.125	1.125	3.22
BC	0.5	0.5	0.25	1.25	3.7
**49**	**PA824**	**SQ**	**TMC**	**ΣFIC**	**$\Delta Log_{10}CFU/ml$**
BS	0.5	0.125	0.25	0.875	1.92
	0.5	0.25	0.125	0.875	1.4
	0.125	0.25	0.5	0.875	1.22
	0.25	0.5	0.125	0.875	1.1
	0.125	0.5	0.25	0.875	1
	0.25	0.5	0.25	1	2.4
	0.5	0.25	0.25	1	2.1
	0.5	0.5	0.125	1.125	2.7
	0.5	0.125	0.5	1.125	2.7
	0.125	0.5	0.5	1.125	2.4
	0.5	0.5	0.25	1.25	3
	0.25	0.5	0.5	1.25	3
	0.5	0.25	0.5	1.25	2.34
BC	0.5	0.5	0.5	1.5	2.7

S. No.	A	B	C	ΣFIC	ΔLog$_{10}$CFU/ml
**50**	**SM**	**SQ**	**THI**	**ΣFIC**	**ΔLog$_{10}$CFU/ml**
BS	0.25	0.125	0.5	0.875	2.7
	0.125	0.5	0.25	0.875	1.19
	0.25	0.5	0.125	0.875	0.91
	0.5	0.25	0.25	1	2.7
	0.25	0.5	0.25	1	1.47
	0.25	0.25	0.5	1	0.95
	0.5	0.5	0.03	1.03	2.7
	0.5	0.5	0.06	1.06	2.4
	0.5	0.06	0.5	1.06	1.7
	0.125	0.5	0.5	1.125	2.7

S. No.	A	B	C	ΣFIC	ΔLog$_{10}$CFU/ml
	0.5	0.125	0.5	1.125	1.55
	0.5	0.5	0.125	1.125	1.55
	0.25	0.5	0.5	1.25	2.7
	0.5	0.25	0.5	1.25	2.4
	0.5	0.5	0.25	1.25	1.7
BC	0.5	0.5	0.5	1.5	2.7
**51**	**SQ**	**THI**	**TMC**	**ΣFIC**	**ΔLog$_{10}$CFU/ml**
BS	0.5	0.25	0.125	0.875	0.92
	0.5	0.25	0.25	1	1.38
	0.25	0.25	0.5	1	0.62
	0.5	0.125	0.5	1.125	2.08
BC	0.5	0.25	0.5	1.25	2.8

Table S3: Steady state exposure (AUC) and Peak concentration (Cmax) observed for a drug in the 3- and 4-drug combination.

Group	Drug	Dose in combination (mg/kg)	C_{max} (µg/ml) in Triplet	AUC_{0-24} (µg.h/ml) in Triplet	C_{max} (µg/ml) in Quartet	AUC_{0-24} (µg.h/ml) in Quartet
1	CFZ	20	2.3±0.2	42.8±5.4	2.4±0.2	48.0±5.2
	BDQ	25	1.6±0.1	21.0±1.7	2.6±0.5	30.9±5.2
	PA824	50	11.2±2.4	76.7±18.5	7.8±1.1	60.9±8.0
	PZA	150	-	-	64.0±9.8	115.4±6.5
2	SQ109	25	5.2±2.7	8.9±3.7	3.5±1.4	5.1±0.8
	BDQ	25	3.6±2.8	34.9±5.8	2.3±0.5	31.4±5.4
	PA824	50	9.3±2.3	86.2±8.6	7.3±1.1	60.7±10.0
	PZA	150	-	-	64.5±3.1	147.5±2.1
3	BTZ043	37.5	2.3±0.9	11.1±5.0	NA	NA
	SQ109	25	0.7±0.5	2.1±0.8	0.5±0.2	1.8±0.4
	MXF	200	2.2±0.9	12.1±3.5	3.9±0.6	13.3±3.1
	PZA	150	-	-	42.0±0.7	155.6±13.2
4	MXF	200	ND**	ND	ND	ND
	THI	800	ND	ND	ND	ND
	PA824	50	ND	ND	ND	ND
	PZA	150	ND	ND	ND	ND
5	EMB	100	1.3±0.6	4.1±1.4	1.2±0.5	4.0±1.1
	BDQ	25	2.0±0.6	22.2±1.6	1.7±0.7	21.5±1.6
	SQ109	25	5.5±5.1	7.8±4.9	2.7±0.5	4.9±0.1
	PZA	150	-	-	67.2±2.5	143.9±29.5
6	BTZ043	37.5	2.1±0.4	15.0±6.2	NA	NA
	THI	800	8.5±2.8	87.0±52.1	5.1±0.4	68.0±16.0
	SQ109	25	0.9±0.5	3.1±0.3	3.1±2.8	4.9±2.2
	PZA	150	-	-	40.2±13.3	153.7±23.8
7	PA824	50	8.4±1.0	133.7±41.7	6.0±0.8	92.1±12.7
	BDQ	25	3.6±0.1	63.2±16.7	4.5±1.3	24.6±2.9
	MXF	400	4.6±0.8	29.8±6.6	5.1±0.7	76.3±13.9
	PZA	150	-	-	33.2±2.3	143.6±16.9
8	PA824	50	13.3±0.5	118.7±13.8	9.8±3.6	66.4±19.0
	BDQ	25	3.1±0.2	43.3±6.0	4.2±1.5	50.7±8.9
	EMB	100	2.7±0.2	9.9±1.6	1.5±0.3	5.7±1.1
	PZA	150	-	-	69.0±12.7	126.9±9.0
9	EMB	100	1.1±0.2	9.6±1.6	NA	NA
	SQ109	25	0.9±0.4	4.7±1.4	3.5±3.1	7.6±3.6
	LZD	130	14.1±0.1	153.5±8.8	14.9±1.2	178.3±12.2
	PZA	150	-	-	39.5±4.4	119.1±7.0
Ref. regimen	INH (H)	30	10.8±2.9	19.8±4.7	7.0±3.2	12.0±3.4
	RIF (R)	10	4.9±1.3	25.4±7.3	5.5±3.0	22.2±8.6
	EMB (E)	100	2.2±0.5	7.9±2.8	1.7±0.6	6.6±1.8
	PZA (Z)	150	-	-	69.7±9.7	160.9±18.8

*NA: data not available due to sample loss

**ND: data not determined in the study, because the group did not complete dosing due to toxicity

Table S4: One way ANOVA with Dunnett's Multiple Comparisons Test. P values marked with asterix are groups which are superior to the reference combination.

**One way ANOVA; Dunnett's Multiple Comparisons Test**		**Mean ΔLog_{10}CFU/lung**	**Significant? $P < 0.05$?**
REFERENCE	COMPARATOR		
INH_RIF_EMB	Pre-treatment Control	-1.8	Yes
	Post-treatment Untreated Control	-1.8	Yes
	CFZ_BDQ_PA824	0.5	No
	SQ109_BDQ_PA824	0.3	No
	BTZ043_SQ109_MXF	0.2	No
	EMB_BDQ_SQ109	1.3	**Yes***
	BTZ043_THI_SQ109	-0.3	No
	PA824_BDQ_MXF	1.7	**Yes***
	PA824_BDQ_EMB	0.3	No
	EMB_SQ109_LZD	-1.0	Yes
INH_RIF_EMB_PZA	Pre-treatment Control	-1.8	Yes
	Post-treatment Untreated Control	-1.9	Yes
	CFZ_BDQ_PA824_PZA	3.1	**Yes***
	SQ109_BDQ_PA824_PZA	3.1	**Yes***
	BTZ043_SQ109_MXF_PZA	1.1	Yes
	EMB_BDQ_SQ109_PZA	3.1	**Yes***
	BTZ043_THI_SQ109_PZA	0.1	No
	PA824_BDQ_MXF_PZA	3.1	**Yes***
	PA824_BDQ_EMB_PZA	3.1	**Yes***
	EMB_SQ109_LZD_PZA	0.7	No
